# Horse Chestnut Saponins–Escins, Isoescins, Transescins, and Desacylescins

**DOI:** 10.3390/molecules28052087

**Published:** 2023-02-23

**Authors:** Philippe Savarino, Emmanuel Colson, Julien André, Pascal Gerbaux

**Affiliations:** Organic Synthesis and Mass Spectrometry Laboratory (S2MOs), University of Mons—UMONS, 23 Place du Parc, 7100 Mons, Belgium

**Keywords:** mass spectrometry, structure–activity relationship, microwave activation, liquid chromatography, hemolytic activity

## Abstract

Escins constitute an abundant family of saponins (saponosides) and are the most active components in *Aesculum hippocastanum* (horse chestnut—HC) seeds. They are of great pharmaceutical interest as a short-term treatment for venous insufficiency. Numerous escin congeners (slightly different compositions), as well as numerous regio-and stereo-isomers, are extractable from HC seeds, making quality control trials mandatory, especially since the structure–activity relationship (SAR) of the escin molecules remains poorly described. In the present study, mass spectrometry, microwave activation, and hemolytic activity assays were used to characterize escin extracts (including a complete quantitative description of the escin congeners and isomers), modify the natural saponins (hydrolysis and transesterification) and measure their cytotoxicity (natural vs. modified escins). The aglycone ester groups characterizing the escin isomers were targeted. A complete quantitative analysis, isomer per isomer, of the weight content in the saponin extracts as well as in the seed dry powder is reported for the first time. An impressive 13% in weight of escins in the dry seeds was measured, confirming that the HC escins must be absolutely considered for high-added value applications, provided that their SAR is established. One of the objectives of this study was to contribute to this development by demonstrating that the aglycone ester functions are mandatory for the toxicity of the escin derivative, and that the cytotoxicity also depends on the relative position of the ester functions on the aglycone.

## 1. Introduction

For decades, pharmaceutical companies and academic laboratories have been engaging in numerous efforts toward the preparation of original molecules with specific properties. Facing huge issues for the low-cost production of safe drugs, scientists have shown increasing interest in molecules of natural origin, presenting highly specific structures, such as drugs or prodrugs. In particular, saponins monopolize an increasing part of current research [1,2]. 

Saponins are specialized metabolites that have been used for hundreds of years in traditional Chinese medicine [3]. Even if these molecules have been discovered in diverse marine invertebrates (e.g., sponges [4], sea stars [5], and sea cucumbers [6]), they are mainly present in the plant kingdom [7]. A study conducted on more than 1700 varieties of Asian plants and vegetables attests to their presence in around 75% of them [8]. This family of molecules, although characterized by an enormous structural diversity, is based on a common pattern, originating from the condensation of an apolar steroidal or triterpenoid aglycone and at least one polar glycan composed of a linear or branched chain of sugar [9]. Monodesmosidic saponins are characterized by the condensation of a single oligosaccharide onto the aglycone, whereas polydesmosidic structures appear when several oligosaccharide chains are linked onto the aglycone [10]. In addition, several specific chemical functions, such as sulfate (either on the glycan [11] or on the aglycone [5]), free carboxylic acid [12], and many others [13,14], are often detected and undoubtedly modulate saponin biological properties. 

In plants and animals, saponins are proposed to be involved in defensive processes, in inter- and intra-species communication, and are also believed to participate in the reproduction processes [15,16,17,18]. Their amphiphilic character gives them interesting and diverse biological properties (e.g., hemolytic, antibacterial, antifungal, antiviral, etc.) [19,20,21,22,23], directly resulting from their specific interactions with cell membranes [24]. It has already been proposed that the cytotoxicity of saponins derives from their ability to be incorporated within cell membranes [2,25,26,27,28]. Of course, their interaction with a specific membrane is highly dependent on their chemical structures, highlighting that the great structural diversity of saponins must be related somehow to specific activities toward the diverse surrounding living organisms. Establishing the structure/activity relationship (SAR) of saponins thus represents a complicated but essential task to decipher the biological role of saponins, but also to envisage saponins as bio-sourced molecules for high-added value applications in pharmacy, medicine, agriculture, etc.

An elegant procedure to meet such an ambitious objective is to use organic chemistry procedures that specifically modify the structure of saponins and evaluate the impact of the modifications on their properties. As typical examples, we recently demonstrated that the desulfation of the saponins contained in the viscera of the Malagasy sea cucumber *Holothuria scabra* cancels their hemolytic activity (HA) [6] and that the partial hydrolysis of the bidesmosidic saponins contained in the husk of the Chilean pseudo-cereal *Chenopodium quinoa* significantly modulates their HA [29]. Other studies have recently been reported and involve the functionalization of the amino group of the diosgenyl β-D-glycosaminosides [30], the *N*-acylation of the *Quillaja saponaria* saponins [31], or the oximation of triterpenoid saponins [32]. 

**Figure 1:**
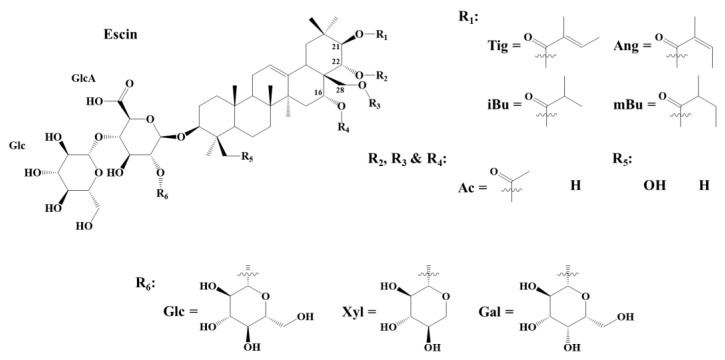
General structure of the escins extracted from the *Aesculus hippocastanum* fruit [33,35,36,37,38,39,40]. Tig, Ang, iBu, mBu, Ac, Glc, Xyl, Gal, and GlcA, stand for tigloyl, angloyl, isobutyryl, 2-methylbutyryl, acetyl, glucose, xylose, galactose, and glucuronic acid, respectively.

The present study focuses on saponins extracted from the fruits of the European horse chestnut, *Aesculus hippocastanum* L. [33]. This tree is widely planted all along the highways or in public parks and gardens, but due to its intrinsic toxicity, seeds (also named conkers) are not suited for human consumption [34]. The fruit, composed of the green spiny husk–the burr–and the seed, thus represents a discarded biomass that contains potentially valuable chemicals, such as a high saponin content. These molecules–named escins–were identified in 1952 by Ratschow M. and Bodecker H. [35]. All the escins are monodesmosidic saponins and consist of a triterpene aglycone and a C3-anchored branched glycan composed of three monosaccharide units (glucose—Glc, xylose—Xyl, galactose—Gal, or glucuronic acid—GlcA) [33,36,37,38,39]. They differ from each other through the presence of specific functions located on carbons C21 (tigloyl—Tig, angloyl—Ang, isobutyryl—iBu, or 2-methylbutyryl—mBu group), C22 or C28 (acetyl group—Ac) [40], as featured in Figure 1. Isomeric escins are mostly distinguished into escins (Tig/Ang at C21; Ac at C22) and isoescins (Tig/Ang at C21; Ac at C28) [33,36,37,38,39]. The literature on escins is quite complicated to follow because various names have appeared over the years, and ambiguities are often present when trying to correlate the different studies. For instance, crypto-, α-, and β-escins appeared in the literature in the 60′s and referred to mixtures of components. Wagner et al. stated that cryptoescin contains C-28-O-acetyl saponins–say isoescins, and β-escin contains C22-O-acetyl saponins, say escins, whereas α-escin is a mixture of crypto- and β-escin [41]. In the recent literature, α-escins and β-escins have mostly been associated with a natural mixture of saponins, with α-escins, and β-escins being mostly composed by Isoescin Ia (a is for tigloyl) and Ib (b is for angloyl), and Escin Ia and Ib, respectively. Commercial preparations available in different countries contain both α- and β-escins, with β-escins being far more active compared to α-escins [42,43,44]. One of the intrinsic reasons for such confusion, besides the presence of isomeric forms in the plants, comes from the fact that α-escins are formed through an acyl migration involving the hydroxyl groups at positions C21, C22, and C28 by simply heating aqueous solutions of β-escins [41,42]. 

In the present study, the ester groups present on C21, on C22, and/or on C28, see Figure 1, of the saponins contained in the conker of the horse chestnut (HC) fruits, will be specifically modified using organic chemistry methods to specifically prepare saponin derivatives possessing potentially modified biological properties. When compared to the glycosidic bonds, the targeted ester functions are indeed prone to hydrolysis under a basic pH, and thus, they can be specifically eliminated under green chemistry conditions, i.e., microwave-assisted activation. The impact of the nature, presence, and position of the ester functions on the cytotoxicity is thus evaluated by measuring their HA. All the samples are qualitatively and quantitatively characterized by mass spectrometry, as abundantly described in the literature [36,37,38,40,45]. Throughout this manuscript, the name of a well-defined molecule will always be written with a capital letter (i.e., Escin Ia and Isoescin IIb), while families of molecules will be written with lowercase letters (typically, escin I and isoescin).

## 2. Results and Discussion

### 2.1. Saponin Extraction and Identification in the Natural Extract (NE)–Purification, Characterization, and Quantification of the Escins I, II and III in the Enriched-Extract (EE)

**Figure 2:**
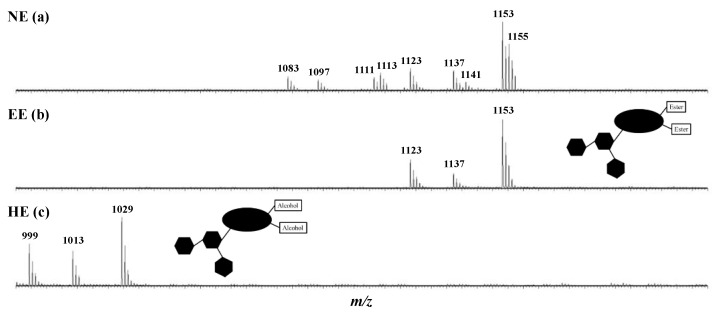
MALDI mass spectra (positive ionization mode) of three different saponin extracts: (**a**) Natural extract (NE); (**b**) Escins I, II and III enriched-extract (EE) obtained by flash chromatography separation (non-polar column–acetonitrile gradient); (**c**) Microwave-assisted alkaline hydrolysis (pH 14–150 °C–5 min) reaction extract (HE). The molecular structures are schematized by highlighting the targeted functions: in EE saponins, the esters are located on C21 (Tig or Ang) and C22 or C28 (Ac); in the HE saponins, the hydrolysis products possess alcohol groups on C21, C22, and C28.

Saponin natural extracts (NE) are obtained by methanol solid–liquid extraction from the ground dry seeds, followed by several liquid/liquid extractions, see Materials and Methods, with an extraction yield of 3.557 g per 25 g of ground dry seed, i.e., 142 mg·g^−1^. Using the MS protocol developed in our laboratory [46], consisting of a compilation of analytical data obtained by matrix-assisted laser desorption/ionization analysis (MALDI), tandem mass spectrometry experiments (collision-induced dissociation—CID), accurate mass measurements (high resolution—HRMS), and liquid chromatography separation (LC-MS/MS), nine different elemental compositions for a total of twenty-four saponin isomers are detected and gathered in Table 1. The MALDI-ToF mass spectrum of the NE is presented in Figure 2 and reveals the presence of nine signals at *m*/*z* 1083, 1097, 1111, 1113, 1123, 1137, 1141, 1153, and 1155 that are readily ascribed to saponin elemental compositions as [M + Na]^+^ ions based on exact mass measurements, see Table 1.

**Figure 3:**
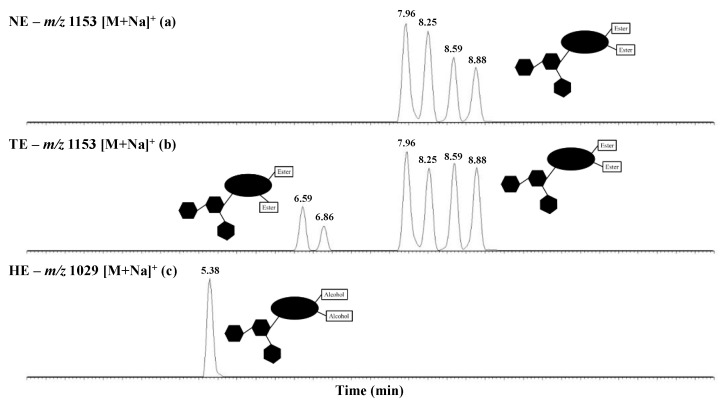
LC mass spectrometry analysis of the escin I derivatives present in the three different saponin extracts: (**a**) The natural extract (NE), *m/z* 1153 EIC (Extract Ion Chromatogram); (**b**) The microwave-assisted transesterification (pH 7–120 °C–5 min) reaction extract (TE), *m/z* 1153 EIC; (**c**) The microwave-assisted alkaline hydrolysis (pH 14–150 °C–5 min) reaction extract (HE), *m/z* 1029 EIC.

LC-MS/MS analyses were performed to (i) confirm that the detected ions were saponin ions, (ii) the presence of isomers for each saponin composition was highlighted, (iii) the glycan sequence and the nature of the ester groups on the aglycone were confirmed using collision-induced dissociations (CID), and (iv) the saponin content was quantified within the NE. LC-MS and LC-MSMS experiments clearly reveal that all the detected escins are present as different isomers, see Table 1, with the typical case of escins I, II, and II present as four different isomers. Based on data from the literature [36,37,38,40] and, in particular, our recent ion mobility mass spectrometry investigation [40], the escins I detected at *m/z* 1153 as [M + Na]^+^ ions in Figure 3a are readily identified as Escin Ia (RT 7.96 min), Escin Ib (RT 8.25 min), Isoescin Ia (RT 8.59 min), and Isoescin Ib (RT 8.88 min). By combining the LC-MS and LC-MS/MS data, we assigned the different retention times (RT) to the different escin molecules. All the CID mass spectra ([M + H]^+^) are presented in Supplementary Materials. Note that all those molecules have already been described in the literature, securing our assignment [33,35,36,37,38,39,40]. 

The saponin molar proportions are estimated based on relative signal ion abundances, i.e., by LC signal integration. As a typical example, in Figure 3a, the extracted ion current (EIC) chromatogram (*m/z* 1153 ions) allows us to estimate–by considering all the detected saponin ions in Table 1–that the four escin I isomers, i.e., Escin Ia, Escin Ib, Isoescin Ia, and Isoescin Ib, are present with molar proportions at 22, 18, 11, and 9%, respectively (Table 1). By using a commercially available saponin (Hederacoside C) as an internal standard, the %-Weight of each escin composition can also be determined, see Table 1, and the escin I composition is confirmed to be the by far most abundant composition, with 55.85% in the weight of the extract, i.e., 55.85 mg of escins I per 100 mg of dry extract, for a global amount of 91.82 mg of escins par 100 mg of dry extract (~92% purity). When combined further with the gravimetric extraction yield, i.e., 142 mg·g^−1^, we can estimate that the escin content in the HC seeds reaches ~130 mg·g^−1^ of dry seed powder, which is in nice agreement with the reported escin contents ~12.5% in HC seeds [47,48,49]. 

We used flash chromatography (FC) to try isolating the escin I isomers and the FC elution, which followed using MALDI-MS. As shown in Figure 2b, the escin I isomers (*m/z* 1153), escin II isomers (*m/z* 1123), and escin III isomers (*m/z* 1137) were successfully separated from escins IV-IX but are too similar for separation from each other. Indeed, escins I, II, and III only differ, isomer per isomer, by the nature of a monosaccharidic residue (R6 in Figure 1), which is glucose, xylose, and galactose, respectively. In other words, the targeted ester groups in C21, C22, or C28 are similar. We thus decided to use such a concentrated extract named here EE for the enriched extract of our investigations.

Using LC-MS with Hederacoside C as the internal standard, we qualitatively and quantitatively analyzed this extract that is composed of the four escin I isomers (27.21%, 21.99%, 19.61%, and 15.99% in weight for Escin Ia, Escin Ib, Isoescin Ia, and Isoescin Ib, respectively), the four escin II isomers (2.75%, 2.48%, 1.18%, and 0.82% in weight for Escin IIa, Escin IIb, Isoescin IIa, and Isoescin IIb, respectively), and the four escin III isomers (2.69%, 2.36%, 1.15%, 0.66% in weight for Escin IIIa, Escin IIIb, Isoescin IIIa, and Isoescin IIIb, respectively), see Table 2. Globally, the EE extract contains 98.89 mg of saponins per 100 mg, with 59.48 mg and 39.41 mg of escins and isoescins per 100 mg, respectively.

### 2.2. Specific Microwave-Assisted Hydrolysis of Horse Chestnut Seed Saponins 

**Figure 4:**
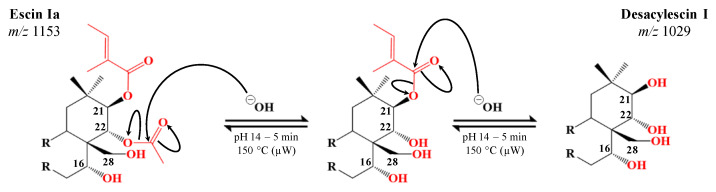
Mechanistic proposal for the successive hydrolysis reactions undergone by escins I, II, and III extracted from the horse chestnut seeds. The hydrolysis corresponds to consecutive acetic acid and angelic (tigloic) acid losses following addition/elimination mechanisms. Note that, in the used hydrolysis conditions, it was not possible to define the hydrolysis sequence if any, since no one-ester-hydrolysis products (either acetic acid or angelic (tigloic) losses) were detected.

One of the objectives of this study was to evaluate whether the ester groups were participating in the cytotoxicity of the saponins. The experimental strategy relies on the specific hydrolysis of these ester functions without degrading the glycan-aglycone escin skeleton. A microwave thermal activation device is well-demonstrated to represent a homogeneous and fast heating method by canceling or limiting the formation of by-products [6,29]. Moreover, the use of microwave heating allows for the modulation of several reaction parameters, such as the concentration of the reagents (here fixed at 1 mg·mL^−1^), the nature of the solvent (here only aqueous solutions), the pH, the heating temperature, and the heating/reaction time [50]. The EE was then dissolved in aqueous buffers ranging from pH 7 to pH 14 (to avoid the glycosidic bond acid hydrolysis) and was subjected to heating times from 1 s to 15 min and heating temperatures from 100 to 160 °C. Using MALDI-MS to monitor the hydrolysis extent, the optimal hydrolysis conditions were identified as pH 14 during 5 min at 150 °C. The MALDI-MS spectrum that was recorded for these conditions is shown in Figure 2c. Escins I, II, and III of the EE are quantitatively hydrolyzed into Desacylescin I, Desacylescin II, and Desacylescin III and detected at *m/z* 1029, *m/z* 999, and *m/z* 1015 in Figure 2c. The ion elemental composition, as well as the conservation of the glycan groups, were confirmed using MALDI-HRMS and LC-MS/MS (see Table 3). The hydrolysis process occurred via an addition/elimination mechanism, which is typical of ester bond basic hydrolysis, as presented in Figure 4.

It can also be noted that the regioisomers and stereoisomers present in each escin family, i.e., escin a, escin b, isoescin a, and isoescin b, collapse into a unique structure, namely desacylescin, due to the elimination of the isomer-determining ester groups. The data shown in Table 3, obtained by qualitative and quantitative analyses, demonstrate the specificity of the ester double hydrolysis since single retention times were obtained. Additionally, the relative proportions between desacylescins I, II, and III (~84, ~8 and ~8%) are shown to reproduce relative proportions between escins I, II, and III (~85, ~7.5 and ~7%) which was determined prior hydrolysis, see Table 2 and Table 3, respectively.

### 2.3. Microwave-Assisted Transesterification of Horse Chestnut Seed Saponins 

**Figure 5:**
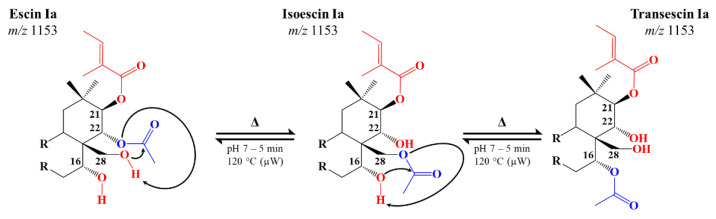
Mechanistic proposal for the successive transesterification reactions undergone by escins I, II, and III extracted from the horse chestnut seed: the first step consists of the displacement of the acetyl group from carbon C22 to carbon C28 (escin to isoescin), and the second step involves the migration of the acetyl group from carbon C28 to C16 (isoescin to transescin).

In addition to escins (a/b) and isoescins (a/b), escin isomers (for escins I, II, and III) were detected upon LC-MS analysis for softer microwave conditions, typically with a neutral pH and lower temperature, as shown in Figure 3b for pH 7–120 °C–5 min. The corresponding saponin mixture is here referred to as the transesterification extract (TE). Based on MALDI-HRMS, the [M + Na]^+^ ion elemental compositions were confirmed to correspond to escin I, II, and III isomers; see Table 4. Escins are well-known to convert into isoescins by an intramolecular acetyl migration–i.e., a transesterification process–from C22 to C28 [51]; see also Figure 5. The comparison between the LC-MS analysis of the EE and TE extracts in Figure 3 reveals that the escin-to-isoescin proportion is modified, with the isoescins becoming more abundant upon heating. Interestingly, it is not possible to increase further the escin-to-isoescin conversion and rather two additional LC signals that appear at lower retention times. From a more quantitative point of view, [51] in the EE, escins (a + b) and isoescins (a + b) are characterized by the %-Weights of ~59.5 and ~39.5 mg per 100 mg; see also Table 2. After microwave thermal activation, this % of weights become, respectively, ~41.5 and ~36.5 mg per 100 mg. The additional escin isomers are then produced toward a %-Weight of ~19.5 mg per 100 mg; see Table 4. Globally, the saponin content in this TE amounts to 97.34 mg per 100 mg; see Table 4. 

As shown in Supplementary Materials, the CID mass spectra of the new escin isomer ions were measured to be marginally distinct from the escin and isoescin CID mass spectra that attested for a great structural similarity. These additional escin isomers may thus correspond to the C-16-O-acetyl saponins. In their 1970 contribution, Wagner et al. managed to crystallize 16-acetyl-21-angeloylprotoascigenin [41], whose escin counterparts have never been reported to the best of our knowledge. We here propose that those Transescins Ia and Ib are produced by an acetyl migration from C28 to C16 that occurs via a 6-membered transition state similar to the C22-to-C28 acetyl migration, as sketched in Figure 5. Such an acetyl migration between C28 to C16 has been described for oleanane derivatives [52]. 

### 2.4. Hemolytic Activity (HA) Evaluation

The cytotoxicity of the different extracts, i.e., enriched-extract (EE), hydrolysis extract (HE), and transesterification extracts (TE), were compared by determining their hemolytic activity (HA) [6,27,29,53,54,55,56,57,58,59,60]. The HA was compared to a referent solution (500 µg·mL^−1^ of *Holothuria scabra* viscera saponins [6]) and expressed as a percentage of the referent solution activity, see Material and Methods. 

**Figure 6:**
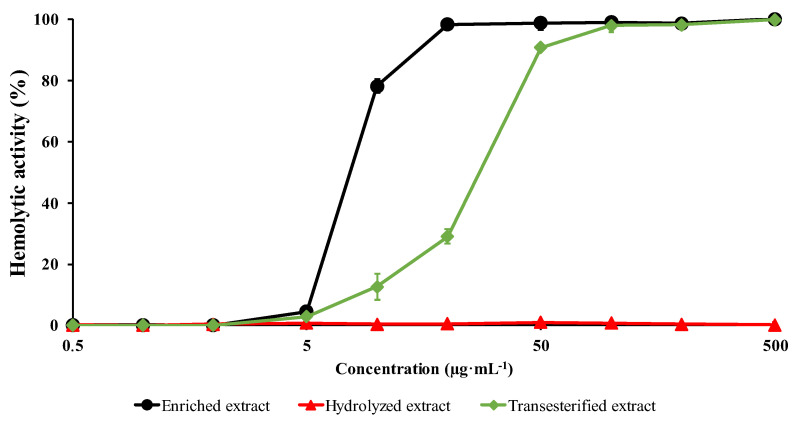
Hemolytic activity of the four saponin extracts: enriched-extract (EE—black line), hydrolyzation extract (HE), and transesterification extract (TE). The experiments were performed using a 2% erythrocytes suspension from bovine blood, in triplicate. HAs are expressed as the % of referent activity, a 500 µg·mL^−1^ of *Holothuria scabra* viscera saponins.

As shown in Figure 6, the HA was significantly impacted by the structural modifications made to the HC saponins. For the EE, the HA reached 100% already at 20 µg·mL^−1^, meaning that the EE saponins had the same cytotoxicity as the standard saponin solution above 20 µg·mL^−1^, making the EE saponins the most active in our study. For a reminder, this extract mostly contains the four escin I isomers (~55.85% in weight) with 59.5% of escins a/b (β-escins) and 39.5% of isoescins a/b (α-escins). Eliminating the aglycone ester functions upon basic hydrolysis clearly cancels the cytotoxicity of the saponins, at least against red blood cells, since HA is no longer detected, whatever the extract concentration: see the red curve in Figure 6. It is likely that such a modification of properties is related to the loss of amphiphilicity of the natural saponins by releasing free alcohol functions on the aglycone. Finally, when estimating the HA of the TE, we observe a loss in activity upon acetyl group migration. We previously determined that the TE was enriched in isoescins (~36% vs. ~39.5% in EE) and transescins (~19.5% vs. 0% in EE) and impoverished in escins (~41.5% vs. ~59.5% in EE) when compared to the EE. These concentrations required us to reach a 100% HA for the EE and TE extracts which are, respectively, determined at ~20 µg·mL^−1^ and ~60 µg·mL^−1^, see Figure 6. When associating these concentrations with the escin isomer proportions, we obtained that 100% HA was reached with EE and TE solutions containing ~10 (escin), ~8 (isoescin), and 0 (transescin) µg·mL^−1^ and ~25 (escin), ~21 (isoescin) and ~11 (transescin) µg·mL^−1^, respectively. It is well-documented that escins (β-escins) are more active than isoescins (α-escins) [42,43,44]. From our data, we must also consider that competitive effects between escin isomers should be considered since, in the presence of greater proportions of isoescins and/or transescins; the concentration of escins must be more than doubled to achieve the same hemolytic activity.

In the recent literature, it is widely reported that the ability of saponins to induce transmembrane pores mostly depends on the saponin structures and the membrane lipid compositions [61]. Cholesterol is often proposed to be the key target of saponins within membranes, and the formation of strong saponin/cholesterol complexes is the driving force of the pore-forming activity of saponins. Therefore, even if the nature of the glycan part is mandatory, i.e., sapogenins are mostly inactive, the structure of the lipophilic part defines the strength of the saponin/cholesterol complexes [62]. Our study nicely correlates with the state-of-the-art knowledge of the saponin SAR since the hydrophobicity of the aglycone part (hydrolysis experiments) and the structure of the aglycone (transesterification experiments) clearly modify the saponin/membrane interactions. In silico investigations and model membrane systems are, nowadays, more and more used for the investigation of interactions between saponins and lipidic membranes [63]. It would be of the utmost interest to consider the present experimental results in such investigations. Indeed, we believe that the measured HA decreases upon hydrolysis and transesterification could find their origin in the reduced penetration propensity of the hydrolyzed saponins in the membrane, on one hand, and in a weaker saponin/cholesterol interaction upon transesterification, on the other hand. 

## 3. Materials and Methods

### 3.1. Chemicals

Technical grade methanol, *n*-hexane, chloroform, dichloromethane, HPLC isobutanol, HPLC grade formic acid, and HPLC grade acetonitrile were provided by CHEM-LAB NV (Somme-Leuze, Belgium). 2,5-dihydroxybenzoic acid (DHB), *N*,*N*-dimethylaniline (DMA), and Hederacoside C were purchased from Sigma-Aldrich (Diegem, Belgium). Sodium citrate, sodium chloride, disodium phosphate dihydrate, potassium chloride, monopotassium phosphate, potassium hydroxide, hydrochloric acid, and borax were provided by VWR Chemicals (Leuven, Belgium). Milli-Q water was produced by purifying tap water with a PURELAB flex 2 (ELGA LabWater, Lane End, High Wycombe, UK). 

### 3.2. Extraction and Flash Chromatography

Mature fruits were collected in Mons (Belgium) during the fall of 2022. Seeds were then separated from the burr, dried in an oven (50 °C overnight), and ground separately (IKA A 11 Mills, IKA, Staufen, Germany). The sample powder was then placed under stirring in methanol (RT overnight). The solution was centrifuged for ten minutes (4500× *g*, Sigma 2-16P, Sigma, Osterode am Harz, Germany). The supernatant was collected and diluted with water to obtain a volume ratio of 70/30 (methanol/water). The methanolic extract was then partitioned (*v*/*v*) with *n*-hexane, chloroform, and dichloromethane to remove apolar compounds. The third methanolic phase was recovered and evaporated under a vacuum with a rotary evaporator (80 rpm, IKA RV 10, IKA, Staufen, Germany) in a water bath (50 °C). The residue was brought to a volume of 25 mL (water), and a fourth liquid/liquid extraction (*v*/*v*) was performed with isobutanol to recover the saponins in the organic phase. The organic phase was then washed twice with water to remove the residual salts and impurities. Finally, the organic phase was evaporated under a vacuum to obtain a purified powder. This extract is named the natural extract in this study (NE).

To separate the escins I, II, and III from other escins, the powder of the natural extract (100 mg) was dissolved in a water/acetonitrile mixture (3 mL, 60/40). The solution was subjected to flash chromatography (Biotage SP1 Flash Chromatography, Biotage Sweden, Uppsala, Sweden) on a non-polar column (Büchi Cartouche FlashPure ID C-18-WP Flash, BUCHI Labortechnik GmbH, Hendrick-Ido-Ambracht, Netherlands) using an eluent whose composition in water and acetonitrile varied during the separation (fraction 1 to 15; 80/20, fraction 16 to 23; 70/30, fraction 24 to 32; 65/35, fraction 33 to 40; 60/40, fraction 41 to 44; 50/50, fraction 45 to 50; from 50/50 to 5/95, and fraction 51 to 63; from 5/95 to 80/20). Each fraction consisted of 18 mL, with a 15 mL·min^−1^ flow. Fractions of interest (36 to 39, confirmed by MALDI-MS) were collected and evaporated under a vacuum. The obtained residue was brought to a volume of 25 mL (water) to carry out a liquid/liquid extraction (*v*/*v*) with isobutanol. The organic phase was washed twice with water and finally evaporated under a vacuum to obtain the purified powder. This extract is named the enriched extract in this study (EE).

### 3.3. Microwave-Assisted Hydrolysis and Transesterification

The hydrolysis and transesterification protocols were adapted from Bedini et al. [64] and from our previous studies [6,29]. *A. hippocastanum* EE (3 mg) was solubilized in 3 mL of different buffer solutions covering a pH range from 7 to 14 [46,50]. pH 7: 50 mL of KH_2_PO_4_ 0.1 mol·L^−1^ were added to 29.1 mL of NaOH 0.1 mol·L^−1^ and brought to 100 mL with Milli-Q water. pH 8: 50 mL of KH_2_PO_4_ 0.1 mol·L^−1^ were added to 46.7 mL of NaOH 0.1 mol·L^−1^ and brought to 100 mL with Milli-Q water. pH 9: 50 mL of borax 0.025 mol·L^−1^ was added to 4.6 mL of HCl 0.1 mol·L^−1^ and brought to 100 mL with Milli-Q water. pH 10: 50 mL of borax 0.025 mol·L^−1^ was added to 18.3 mL of NaOH 0.1 mol·L^−1^ and brought to 100 mL with Milli-Q water. pH 11: 50 mL of Na_2_HPO_4_ 0.05 mol·L^−1^ were added to 4.1 mL of NaOH 0.1 mol·L^−1^ and brought to 100 mL with Milli-Q water. pH 12: 50 mL of Na_2_HPO_4_ 0.05 mol·L^−1^ were added to 26.9 mL NaOH 0.1 mol·L^−1^ and brought to 100 mL with Milli-Q water. pH 13: 4 mg of NaOH were dissolved in 100 mL Milli-Q water. pH 14: 4 g of NaOH were dissolved in 100 mL Milli-Q water. 

The different samples were subjected to different times of heating (1 s to 15 min) at several temperatures (from 100 °C to 160 °C) using a microwave device (Biotage Initiator Classic, Biotage Sweden, Uppsala, Sweden). After cooling to room temperature, the solutions were brought to 7 using HCl (0.1 mol·L^−1^), and extraction was performed against isobutanol (*v*/*v*). The organic phase was washed twice with water and finally evaporated under a vacuum to produce a dry powder. These extracts are named the hydrolyzed extract (HE) and transesterification extract (TE) in this study, see text.

### 3.4. Mass Spectrometry (MS) Analyses

Preliminary MS analyses were performed using a matrix-assisted laser desorption/ionization (MALDI) and a Waters QToF Premier mass spectrometer (Waters, Manchester, UK) in the positive ionization mode. The matrix was composed of a mixture of DHB (25 mg) and DMA (6 µL, 99%) in 250 µL of Milli-Q water/acetonitrile (*v*/*v*). A matrix droplet (1 µL) was placed on a stainless-steel plate and air-dried. A sample solution (1 mg of sample dissolved in 1 mL of Milli-Q water/acetonitrile (*v*/*v*)) droplet (1 µL) was spotted on the top of the matrix crystal and air-dried before introducing the plate into the mass spectrometer source. The MALDI source was composed of an Nd-YAG laser with a maximum energy of 104.1 µJ, which was transferred to the sample in a 2.2 ns pulse (200 Hz). The quadrupole was configured in the rf-only mode to let the ions pass between *m/z* 250 and 2000. All the ions were mass-analyzed using the ToF analyzer (reflectron mode, resolution around 10,000) with 1 s of integration time. Accurate mass measurements (HRMS) were performed using PEG 600-1500 as an external standard (lock mass). For the MALDI-MSMS experiments, precursor ions were mass-selected by quadrupole and collided against argon in the collision cell, with the kinetic energy of the laboratory frame selected to afford an intense enough production of ion signals. The fragment ions were mass-measured with the ToF analyzer (1 s of integration time).

Liquid chromatography MS (LC-MS) analyses were carried out with a Waters Acquity UPLC H-Class (Waters, Manchester, UK) composed of a vacuum degasser, a quaternary pump, and an autosampler, coupled to a Waters Synapt G2-S*i* mass spectrometer (Waters, Manchester, UK), with a non-polar column (Acquity UPLC BEH C18; 2.1 × 50 mm; 1.7 µm; Waters, Manchester, UK) used at 40 °C. Samples (0.1 mg) were dissolved (1 mL) in a Milli-Q water/acetonitrile (85/15) solution, and a volume of 5 µL (sample room at 10 °C) was injected into the column. The gradient optimized for the saponins followed a flow rate of 250 µL·mL^−1^ of Milli-Q water (with 0.1% formic acid, eluent A) and acetonitrile (eluent B). Elution started with 85% eluent A and 15% eluent B, reached 60% of eluent A and 40% eluent B at 6 min and maintained for 3 min. The ratio was modified to reach 5% eluent A and 95% eluent B in 2 min, maintained for 1 min, brought back to 85% eluent A and 15% eluent B in 1 min, and finally maintained for 2 min, until the end of the chromatographic run (15 min in all). Electrospray ionization (ESI, positive mode) was used for the saponin ion production with the following conditions: capillary voltage 3.1 kV, cone voltage 40 V; source offset 80 V, source temperature 100 °C, desolvation temperature 300 °C (dry nitrogen flow rate 500 L·h^−1^), and mass range between *m/z* 50 and 2000 (quadrupole in rf-only mode, resolution around 22,000) with 1 s of integration time. For LC-MSMS analyses, precursor ions were mass-selected by the quadrupole and collided against argon in the Trap cell of the Tri-Wave device, with the kinetic energy of the laboratory frame selected to afford intense enough production signals. The fragment ions were mass-measured with the ToF analyzer (1 s of integration time).

The quantification of NE and EE was achieved by adding a known quantity (0.1 mg·mL^−1^) of commercially available Hederacoside C (Sigma-Aldrich, Product n° 97151, M-Clarity^TM^ Program MQ100), a pure saponin extracted from *Hedera helix*, as the internal standard in each extract. The spiked samples were analyzed by LC-MS using the experimental conditions described above. For each saponin, including Hederacoside C, the corresponding LC-MS ion signals were integrated using the integration algorithm, which is available under MassLynx^TM^ 4.1 Software, including all the isotopic compositions. The global ion counts were used to estimate each saponin concentration comparatively for Hederacoside C signal integration. The %-Weights in NE and EE (see Table 1 and Table 2, respectively) corresponded to the mass percentages of saponins with a given elemental composition within the extract (please note that the sums of the %-Weight do not achieve 100%, making it possible to estimate the saponin content within the extracts at 91.82% and 98.89% for NE and EE, respectively). The mass fractions in seed expressed in mg·g^−1^ were further calculated by using the global yield of extraction determined at 142 mg of extract per g of ground seed (NE). 

### 3.5. Hemolytic Activity (HA) Measurements

To measure the HA, which reflects the membranolytic activity, bovine blood (stored with 0.11 M sodium citrate) was collected immediately after animal death at the “Abattoirs de Ath” (22 Chemins des Peupliers, 7800 Ath, Belgium) on 4 February 2022. The bovine blood was washed using a phosphate-buffered saline (PBS) solution, which was prepared by dissolving 8 g of NaCl, 1.45 g of Na_2_HPO_4_·2H_2_O, 0.2 g of KCl and 0.2 g KH_2_PO_4_ in 800 mL of Milli-Q water adjusting the pH of the solution to 7.4 and bringing the solution to a volume of 1 L, using Milli-Q water. In a 50 mL Falcon, 10 mL of citrated bovine blood was added to 40 mL of the PBS solution. The Falcon was centrifuged (15 min, 10,000× *g*), and the pellet was preserved and washed again using PBS solution until a clear and colorless supernatant was obtained. The final supernatant was discarded, and 2 mL from the pellet was diluted with 98 mL of PBS solution, giving a 2% (*v*/*v*) erythrocytes suspension. Various solutions containing saponin extracts at different concentrations were prepared. These solutions were placed in the presence of the 2% red blood cells suspension in triplicate and incubated for 60 min at 20 °C, continuously under shaking (500 rpm), before being centrifuged (10 min, 10,000× *g*). The supernatant of each sample was collected to measure the absorbance of the solution at 540 nm [65]. In these experiments, we used a 500 µg·mL^−1^ solution of saponins extracted from *Holothuria scabra* viscera as a reference solution, which is extremely membranolytic [6]. The HAs of the extracts were calculated using the following equation:

(1) $$\mathrm{HA}\;\left(\%\right)=\frac{\left({\mathrm{Abs}}_{\mathrm{sample}}-{\mathrm{Abs}}_{\mathrm{blank}}\right)}{\left({\mathrm{Abs}}_{\mathrm{ref}}-{\mathrm{Abs}}_{\mathrm{blank}}\right)}*100$$

where Abs_sample_, Abs_blank_, and Abs_ref_ corresponded to the absorbance (540 nm) of the tested erythrocytes/saponins solutions, of the erythrocyte solution and of the erythrocyte/referent saponin solution, respectively [6,29,46,65]. 

## 4. Conclusions

Escins and isoescins from the *Aesculus hippocastanum* fruit are well-known to participate in the toxicity of the seeds. In the present study, we studied the influence of nature as well as the position of the aglycone ester groups on the cytotoxicity of the escins. We used mass spectrometry, microwave heating, and hemolytic activity on extracted saponins to, respectively, characterize the saponin extracts, chemically modify the saponins, and probe their cytotoxicity.

We fully characterized the saponin natural extract by providing, for the first time, for all the detected isomers, their weight percentages in the seeds (and in the extract) with an impressive bioavailability of 13% in weight for the saponins in the dry seed powder. We successfully prepared an extract that constituted 99% in weight of the escins I, II, and III using flash chromatography, with a complete qualitative and quantitative description of the regio- and stereoisomer content. Using microwave heating at pH 14 for 5 min, we successfully hydrolyzed the glycan ester groups yielding a fully characterized mixture of deacylescins I, II, and III. We also demonstrated that upon microwave heating at pH 7, the escin hydrolysis was replaced by successive transesterification processes corresponding to the acetic acid residue transfers from C22 (escins) to C28 (isoescins) and C28 to C16 (transescins). These transescins could correspond at least partly to the cryoescins described in the 70′s. From the escin extract (99% in escins; ~60% in escins; ~40% in isoescins), a saponin mixture rich in isoescins and transescins was produced (97% in escins; ~40% in escins; ~35% in isoescins; ~20% in transecins). 

The hemolytic activities (HA) of these three extracts (enriched-extract—EE; hydrolyzed-extract—HE; transesterification-extract—TE) were shown to be drastically impacted by the structural modifications. The EE was measured as the most hemolytic saponin mixture, whereas the full hydrolysis of the aglycone ester groups was shown to cancel the HA. The HA of the TE was shown to suffer a three-fold decrease when compared to the escin(I-II-III)-enriched extract, i.e., 100% HA was reached with EE and TE solutions containing ~10 (escin), ~8 (isoescin), and 0 (transescin) µg·mL^−1^ and ~25 (escin), ~21 (isoescin), and ~11 (transescin) µg·mL^−1^, respectively. Based on the literature, escins are known to be more cytotoxic than their isoescin isomers [44]. However, since the concentration of the most active escins drastically increased, from 10 to 25 µg·mL^−1^, we believe that competitive effects between escin isomers must be considered since, in the presence of greater proportions of isoescins and/or transescins, the concentration of escins must be more than doubled to achieve the same hemolytic activity. Further experiments are obviously expected to confirm such a hypothesis and will absolutely have to pass through the separation of the three regioisomers. This task promises to be particularly delicate in view of the great structural similarities. 

Nevertheless, this study clearly reveals the impact of the nature, presence, and position of the aglycone ester functions on the biological activity of the escin isomers. 

## Figures and Tables

**Table 1:** *Aesculus hippocastanum* seed extract: data collected by MS-based experiments. Compositions and mass error measurements (Δ) were determined by MALDI-HRMS. The %-Weights in extracts and mass fractions (mg·g^−1^ of *Aesculus hippocastanum* seed powder) of each saponin composition were determined based on the LC ion signal intensity ratios, using Hederacoside C as the internal standard, and using the gravimetric extraction yield (142 mg·g^−1^). The saponin molar proportions (%) were determined based on the LC ion signal relative integration. See Materials and Methods for the details of all quantitative analysis.

Saponins	Elemental Compositions (M)	*m/z* (Δ ppm) [M + Na]^+^	R_1_	R_2_	R_3_	R_4_	R_5_	R_6_	%-Weights in Extract (%)	Mass Fractions in Seed (mg·g^−1^)	Retention Time (min)	Molar Proportions (%)
Escin Ia	C_55_H_86_O_24_	1153.5427 (1.7)	Tig	Ac	H	H	OH	Glc	55.85 ± 0.03	79.46	7.96	21.72 ± 0.09
Escin Ib			Ang	Ac	H	H	OH	Glc			8.25	18.40 ± 0.08
Isoescin Ia			Tig	H	Ac	H	OH	Glc			8.59	10.77 ± 0.08
Isoescin Ib			Ang	H	Ac	H	OH	Glc			8.88	9.32 ± 0.14
Escin IIa	C_54_H_84_O_23_	1123.5342 (3.6)	Tig	Ac	H	H	OH	Xyl	4.18 ± 0.11	5.95	7.98	1.48 ± 0.09
Escin IIb			Ang	Ac	H	H	OH	Xyl			8.28	1.26 ± 0.07
Isoescin IIa			Tig	H	Ac	H	OH	Xyl			8.66	0.93 ± 0.12
Isoescin IIb			Ang	H	Ac	H	OH	Xyl			8.94	0.99 ± 0.09
Escin IIIa	C_55_H_86_O_23_	1137.5453 (0.4)	Tig	Ac	H	H	H	Gal	4.74 ± 0.08	6.75	8.45	1.96 ± 0.08
Escin IIIb			Ang	Ac	H	H	H	Gal			8.84	1.66 ± 0.06
Isoescin IIIa			Tig	H	Ac	H	H	Gal			9.18	0.82 ± 0.03
Isoescin IIIb			Ang	H	Ac	H	H	Gal			9.54	0.75 ± 0.04
Escin IV	C_52_H_82_O_24_	1113.5134 (3.6)	Ac	Ac	H	H	OH	Glc	15.22 ± 0.16	21.66	6.10	9.16 ± 0.26
Isoescin IV			Ac	H	Ac	H	OH	Glc			6.56	7.84 ± 0.17
Escin V	C_54_H_86_O_24_	1141.5435 (2.6)	iBu	Ac	H	H	OH	Glc	7.27 ± 0.21	10.34	7.71	6.81 ± 0.15
Isoescin V			iBu	H	Ac	H	OH	Glc			8.20	1.11 ± 0.24
Escin VI	C_55_H_88_O_24_	1155.5537 (2.3)	mBu	Ac	H	H	OH	Glc	2.47 ± 0.17	3.52	8.62	2.18 ± 0.01
Isoescin VI			mBu	H	Ac	H	OH	Glc			9.47	0.48 ± 0.05
Escin VII	C_51_H_80_O_23_	1083.5001 (1.2)	Ac	Ac	H	H	OH	Xyl	0.56 ± 0.10	0.79	6.12	0.31 ± 0.09
Isoescin VII			Ac	H	Ac	H	OH	Xyl			6.59	0.33 ± 0.06
Escin VIII	C_53_H_84_O_23_	1111.5316 (1.3)	iBu	Ac	H	H	OH	Xyl	0.78 ± 0.13	1.11	7.42	0.54 ± 0.03
Isoescin VIII			iBu	H	Ac	H	OH	Xyl			7.64	0.33 ± 0.04
Escin IX	C_52_H_82_O_23_	1097.5182 (3.4)	Ac	Ac	H	H	H	Glc	0.75 ± 0.17	1.07	6.44	0.46 ± 0.05
Isoescin IX			Ac	H	Ac	H	H	Glc			6.83	0.39 ± 0.07

**Table 2:** Flash chromatography separation (non-polar column–acetonitrile solvent) of *Aesculus hippocastanum* seed saponin extract given the escin I, II and III enriched-extract: the ion elemental compositions were determined by MALDI-HRMS, the composition molar proportion (%) of each escin composition, the %-Weights in EE (%), the retention time (min), and the isomer molar proportion (%) were estimated based on the integration of the signals obtained by LC-MS, and using Hederacoside C as the internal standard.

Elemental Compositions (M)	*m/z* (Δ ppm) [M + Na]^+^	Isomer	Retention Time (min)	Molar Proportion (%)	%-Weights in Extract (%)	Isomer Molar Proportions (%)
Escin I C_55_H_86_O_24_	1153.5427 (1.7)	Escin Ia	7.96	85.20 ± 0.09	27.21 ± 0.05	32.06 ± 0.04
		Escin Ib	8.25		21.99 ± 0.09	25.92 ± 0.07
		Isoescin Ia	8.59		19.61 ± 0.07	23.13 ± 0.06
		Isoescin Ib	8.88		15.99 ± 0.11	18.89 ± 0.09
Escin II C_54_H_84_O_23_	1123.5342 (3.6)	Escin IIa	7.98	7.53 ± 0.12	2.75 ± 0.09	37.89 ± 0.08
		Escin IIb	8.28		2.48 ± 0.06	34.26 ± 0.04
		Isoescin IIa	8.66		1.18 ± 0.07	16.94 ± 0.05
		Isoescin IIb	8.94		0.82 ± 0.04	10.91 ± 0.03
Escin III C_55_H_86_O_23_	1137.5453 (0.4)	Escin IIIa	8.45	7.26 ± 0.09	2.69 ± 0.06	38.43 ± 0.05
		Escin IIIb	8.84		2.36 ± 0.09	33.92 ± 0.04
		Isoescin IIIa	9.18		1.15 ± 0.05	17.20 ± 0.03
		Isoescin IIIb	9.54		0.66 ± 0.02	10.45 ± 0.02

**Table 3:** Microwave-assisted alkaline hydrolysis (pH 14–150 °C–5 min) of *Aesculus hippocastanum* seed escin I, II, and III enriched-extract: the ion elemental compositions were determined by MALDI-HRMS, and the molar proportions (%) and retention times (min) were obtained by LC-MS.

Elemental Compositions (M)	*m/z* (Δ ppm) [M + Na]^+^	R_1_	R_2_	R_3_	R_4_	R_5_	R_6_	Retention Time (min)	Molar Proportion (%)
Desacylescin I C_48_H_78_O_22_	1029.4876 (0.7)	H	H	H	H	OH	Glc	5.38	83.71 ± 0.48
Desacylescin II C_47_H_76_O_21_	999.4745 (3.2)	H	H	H	H	OH	Xyl	5.36	8.15 ± 0.06
Desacylescin III C_48_H_78_O_21_	1013.4953 (2.0)	H	H	H	H	H	Gal	5.39	8.14 ± 0.51

**Table 4:** Microwave-assisted alkaline transesterification (pH 7–120 °C–5 min) of *Aesculus hippocastanum* seed enriched-extract: ion elemental compositions were determined by MALDI-HRMS, and the molar proportion (%) were obtained based on LC-MS signal integration.

Saponins	Elemental Compositions (M)	*m/z* (Δ ppm) [M + Na]^+^	R_1_	R_2_	R_3_	R_4_	R_5_	R_6_	Retention Time (min)	Composition Molar Proportions (%)	%-Weights in Extract (%)	Isomer Molar Proportions (%)
Escin Ia	C_55_H_86_O_24_	1153.5427 (1.7)	Tig	Ac	H	H	OH	Glc	7.96	84.44 ± 0.18	19.91 ± 0.09	23.93 ± 0.17
Escin Ib			Ang	Ac	H	H	OH	Glc	8.25		15.80 ± 0.10	18.99 ± 0.23
Isoescin Ia			Tig	H	Ac	H	OH	Glc	8.59		16.53 ± 0.08	19.89 ± 0.15
Isoescin Ib			Ang	H	Ac	H	OH	Glc	8.88		14.63 ± 0.06	17.59 ± 0.21
Transescin Ia			Tig	H	H	Ac	OH	Glc	6.59		9.22 ± 0.03	11.08 ± 0.13
Transescin Ib			Ang	H	H	Ac	OH	Glc	6.86		7.10 ± 0.09	8.53 ± 0.21
Escin IIa	C_54_H_84_O_23_	1123.5342 (3.6)	Tig	Ac	H	H	OH	Xyl	7.98	8.72 ± 0.23	1.68 ± 0.08	21.28 ± 0.26
Escin IIb			Ang	Ac	H	H	OH	Xyl	8.28		1.48 ± 0.07	18.72 ± 0.50
Isoescin IIa			Tig	H	Ac	H	OH	Xyl	8.66		1.58 ± 0.08	19.98 ± 0.45
Isoescin IIb			Ang	H	Ac	H	OH	Xyl	8.94		1.57 ± 0.07	19.83 ± 0.68
Transescin IIa			Tig	H	H	Ac	OH	Xyl	6.58		1.01 ± 0.01	12.84 ± 0.24
Transescin IIb			Ang	H	H	Ac	OH	Xyl	6.85		0.69 ± 0.06	8.79 ± 0.27
Escin IIa	C_55_H_86_O_23_	1137.5453 (0.4)	Tig	Ac	H	H	H	Gal	8.45	6.84 ± 0.42	1.38 ± 0.08	22.53 ± 0.26
Escin IIb			Ang	Ac	H	H	H	Gal	8.84		1.13 ± 0.03	18.43 ± 0.73
Isoescin IIa			Tig	H	Ac	H	H	Gal	9.18		1.21 ± 0.07	19.69 ± 0.69
Isoescin IIb			Ang	H	Ac	H	H	Gal	9.54		1.10 ± 0.08	17.91 ± 0.20
Transescin IIa			Tig	H	H	Ac	H	Gal	7.00		0.72 ± 0.02	11.73 ± 0.28
Transescin IIb			Ang	H	H	Ac	H	Gal	7.27		0.60 ± 0.03	9.71 ± 0.37

## Data Availability

The data presented in this study are available on request from the corresponding author.

## Supplementary Materials

The following supporting information can be downloaded at: https://www.mdpi.com/article/10.3390/molecules28052087/s1. Figure S1: LC-MSMS(+) analysis of Escins I, II and III enriched-extract (EE): CID spectrum (15 eV) recorded for the *m/z* 1131 precursor ions [M + H]+ at 7.96 min retention time (Escin Ia); Figure S2: LC-MSMS(+) analysis of Escins I, II and III enriched-extract (EE): CID spectrum (15 eV) recorded for the *m/z* 1131 precursor ions [M + H]+ at 8.25 min retention time (Escin Ib); Figure S3: LC-MSMS(+) analysis of Escins I, II and III enriched-extract (EE): CID spectrum (15 eV) recorded for the *m/z* 1131 precursor ions [M + H]+ at 8.59 min retention time (Isoescin Ia); Figure S4: LC-MSMS(+) analysis of Escins I, II and III enriched-extract (EE): CID spectrum (15 eV) recorded for the *m/z* 1131 precursor ions [M + H]+ at 8.88 min retention time (Isoescin Ib); Figure S5: LC-MSMS(+) analysis of Transesterification extract (TE): CID spectrum (15 eV) recorded for the *m/z* 1131 precursor ions [M + H]+ at 6.59 min retention time (Transescin Ia); Figure S6: LC-MSMS(+) analysis of Transesterification extract (TE): CID spectrum (15 eV) recorded for the *m/z* 1131 precursor ions [M + H]+ at 6.86 min retention time (Transescin Ib); Figure S7: LC-MSMS(+) analysis of Hydrolysis extract (HE): CID spectrum (15 eV) recorded for the *m/z* 1007 precursor ions [M + H]+ at 5.38 min retention time (Desacylescin I); Figure S8: LC-MSMS(+) analysis of Escins I, II and III enriched-extract (EE): CID spectrum (15 eV) recorded for the *m/z* 1101 precursor ions [M + H]+ at 7.98 min retention time (Escin IIa); Figure S9: LC-MSMS(+) analysis of Escins I, II and III enriched-extract (EE): CID spectrum (15 eV) recorded for the *m/z* 1101 precursor ions [M + H]+ at 8.28 min retention time (Escin IIb); Figure S10: LC-MSMS(+) analysis of Escins I, II and III enriched-extract (EE): CID spectrum (15 eV) recorded for the *m/z* 1101 precursor ions [M + H]+ at 8.66 min retention time (Isoescin IIa); Figure S11: LC-MSMS(+) analysis of Escins I, II and III enriched-extract (EE): CID spectrum (15 eV) recorded for the *m/z* 1101 precursor ions [M + H]+ at 8.94 min retention time (Isoescin IIb); Figure S12: LC-MSMS(+) analysis of Transesterification extract (TE): CID spectrum (15 eV) recorded for the *m/z* 1101 precursor ions [M + H]+ at 6.58 min retention time (Transescin IIa); Figure S13: LC-MSMS(+) analysis of Transesterification extract (TE): CID spectrum (15 eV) recorded for the *m/z* 1101 precursor ions [M + H]+ at 6.85 min retention time (Transescin IIb); Figure S14: LC-MSMS(+) analysis of Hydrolysis extract (HE): CID spectrum (15 eV) recorded for the *m/z* 977 precursor ions [M + H]+ at 5.36 min retention time (Desacylescin II); Figure S15: LC-MSMS(+) analysis of Escins I, II and III enriched-extract (EE): CID spectrum (15 eV) recorded for the *m/z* 1115 precursor ions [M + H]+ at 8.45 min retention time (Escin IIIa); Figure S16: LC-MSMS(+) analysis of Escins I, II and III enriched-extract (EE): CID spectrum (15 eV) recorded for the *m/z* 1115 precursor ions [M + H]+ at 8.84 min retention time (Escin IIIb); Figure S17: LC-MSMS(+) analysis of Escins I, II and III enriched-extract (EE): CID spectrum (15 eV) recorded for the *m/z* 1115 precursor ions [M + H]+ at 9.18 min retention time (Isoescin IIIa); Figure S18: LC-MSMS(+) analysis of Escins I, II and III enriched-extract (EE): CID spectrum (15 eV) recorded for the *m/z* 1115 precursor ions [M + H]+ at 9.54 min retention time (Isoescin IIIb); Figure S19: LC-MSMS(+) analysis of Transesterification extract (TE): CID spectrum (15 eV) recorded for the *m/z* 1115 precursor ions [M + H]+ at 7.00 min retention time (Transescin IIIa); Figure S20: LC-MSMS(+) analysis of Transesterification extract (TE): CID spectrum (15 eV) recorded for the *m/z* 1115 precursor ions [M + H]+ at 7.27 min retention time (Transescin IIIb); Figure S21: LC-MSMS(+) analysis of Hydrolysis extract (HE): CID spectrum (15 eV) recorded for the *m/z* 991 precursor ions [M + H]+ at 5.39 min retention time (Desacylescin III); Figure S22: LC-MSMS(+) analysis of Natural extract (NE): CID spectrum (15 eV) recorded for the *m/z* 1091 precursor ions [M + H]+ at 6.10 min retention time (Escin IV); Figure S23: LC-MSMS(+) analysis of Natural extract (NE): CID spectrum (15 eV) recorded for the *m/z* 1091 precursor ions [M + H]+ at 6.56 min retention time (Isoescin IV); Figure S24: LC-MSMS(+) analysis of Natural extract (NE): CID spectrum (15 eV) recorded for the *m/z* 1119 precursor ions [M + H]+ at 7.71 min retention time (Escin V); Figure S25: LC-MSMS(+) analysis of Natural extract (NE): CID spectrum (15 eV) recorded for the *m/z* 1119 precursor ions [M + H]+ at 8.20 min retention time (Isoescin V); Figure S26: LC-MSMS(+) analysis of Natural extract (NE): CID spectrum (15 eV) recorded for the *m/z* 1133 precursor ions [M + H]+ at 8.62 min retention time (Escin VI); Figure S27: LC-MSMS(+) analysis of Natural extract (NE): CID spectrum (15 eV) recorded for the *m/z* 1133 precursor ions [M + H]+ at 9.47 min retention time (Isoescin VI); Figure S28: LC-MSMS(+) analysis of Natural extract (NE): CID spectrum (15 eV) recorded for the *m/z* 1061 precursor ions [M + H]+ at 6.12 min retention time (Escin VII); Figure S29: LC-MSMS(+) analysis of Natural extract (NE): CID spectrum (15 eV) recorded for the *m/z* 1061 precursor ions [M + H]+ at 6.59 min retention time (Isoescin VII); Figure S30: LC-MSMS(+) analysis of Natural extract (NE): CID spectrum (15 eV) recorded for the *m/z* 1089 precursor ions [M + H]+ at 7.42 min retention time (Escin VIII); Figure S31: LC-MSMS(+) analysis of Natural extract (NE): CID spectrum (15 eV) recorded for the *m/z* 1089 precursor ions [M + H]+ at 7.64 min retention time (Isoescin VIII); Figure S32: LC-MSMS(+) analysis of Natural extract (NE): CID spectrum (15 eV) recorded for the *m/z* 1075 precursor ions [M + H]+ at 6.44 min retention time (Escin IX); Figure S33: LC-MSMS(+) analysis of Natural extract (NE): CID spectrum (15 eV) recorded for the *m/z* 1075 precursor ions [M + H]+ at 6.83 min retention time (Isoescin IX).

## Sample Availability

Samples of the compounds are available from the authors upon request.
