# Supplementary material for: Horse Chestnut Saponins–Escins, Isoescins, Transescins, and Desacylescins
Source: Molecules. 2023 Feb 23;28(5):2087. doi: 10.3390/molecules28052087 (PMC10004172; doi:10.3390/molecules28052087)
Supplement: Supplementary file 1 [file molecules-28-02087-s001.zip › molecules-2224658-supplementary.pdf]

Horse Chestnut saponins – Escins, Isoescins, Transescins, and Desacylescins

Philippe Savarino, Emmanuel Colson, Julien André, and Pascal Gerbaux *

Organic Synthesis and Mass Spectrometry Laboratory (S²MOs), University of Mons – UMONS, 23 Place du Parc, 7100 Mons, Belgium; philippe.savarino@umons.ac.be (P.S.); colson-emmanuel@outlook.be (E.C.); ailurus.jul@gmail.com (J.A.)

* Correspondence: pascal.gerbaux@umons.ac.be (P.G.)

Supporting information

Escin Ia (7.96 min)

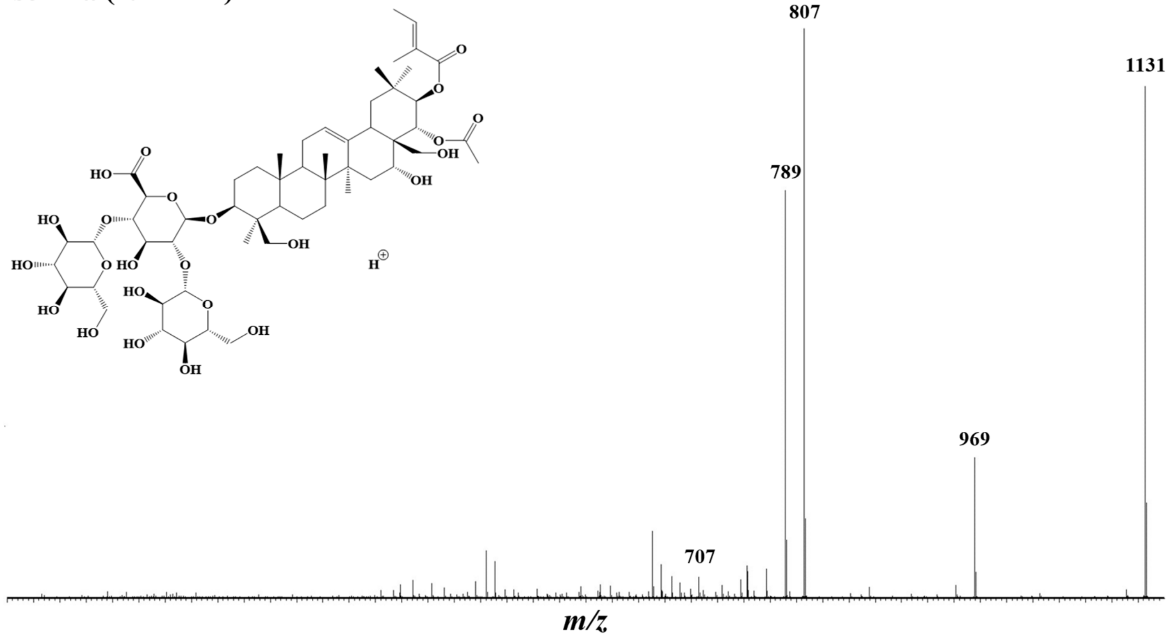

Figure S1: LC-MSMS(+) analysis of Escins I, II and III enriched-extract (EE): CID spectrum (15 eV) recorded for the *m/z* 1131 precursor ions $[M+H]^+$ at 7.96 min retention time (Escin Ia)

Escin Ib (8.25 min)

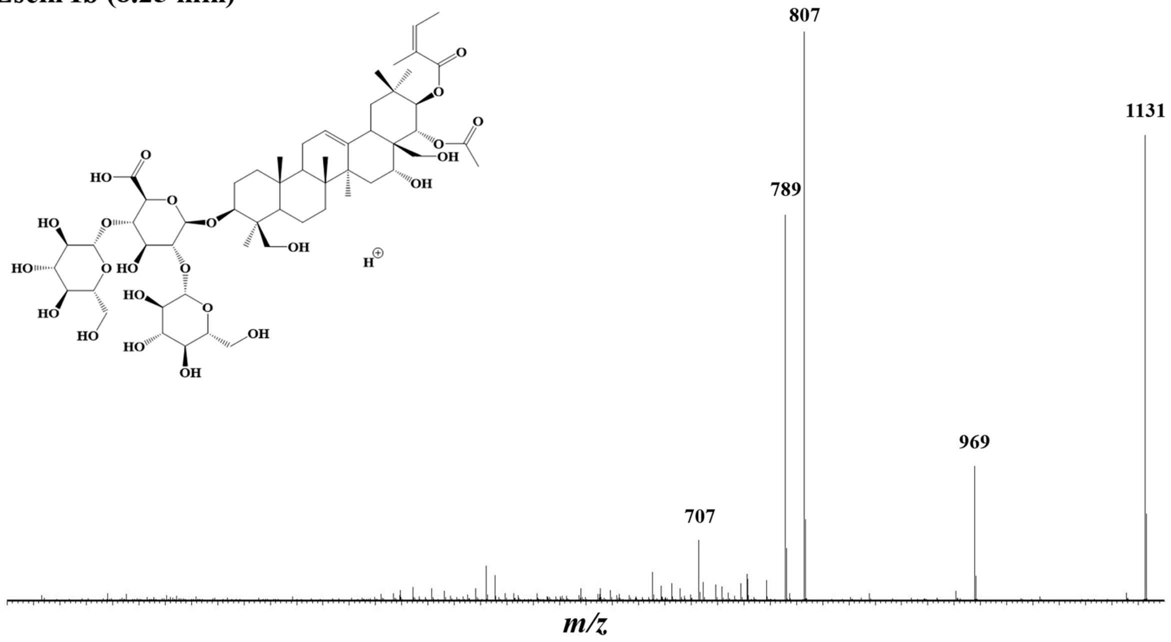

Figure S2: LC-MSMS(+) analysis of Escins I, II and III enriched-extract (EE): CID spectrum (15 eV) recorded for the *m/z* 1131 precursor ions $[M+H]^+$ at 8.25 min retention time (Escin Ib)

Isoescin Ia (8.59 min)

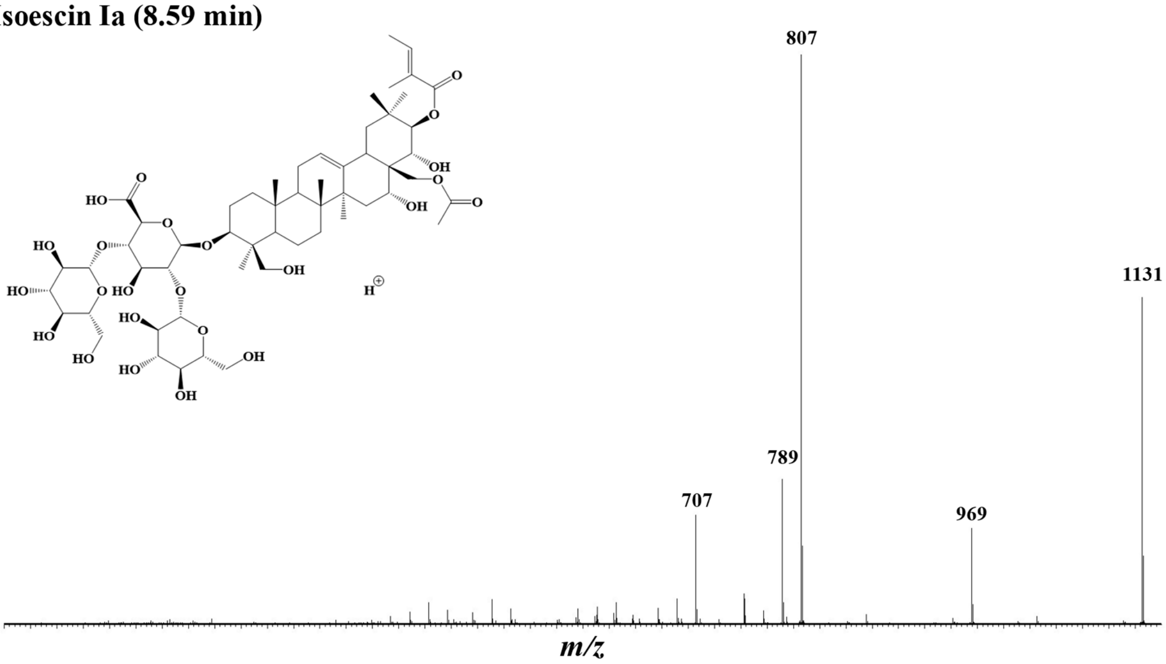

Figure S3: LC-MSMS(+) analysis of Escins I, II and III enriched-extract (EE): CID spectrum (15 eV) recorded for the *m/z* 1131 precursor ions $[M+H]^+$ at 8.59 min retention time (Isoescin Ia)

Isoescin Ib (8.88 min)

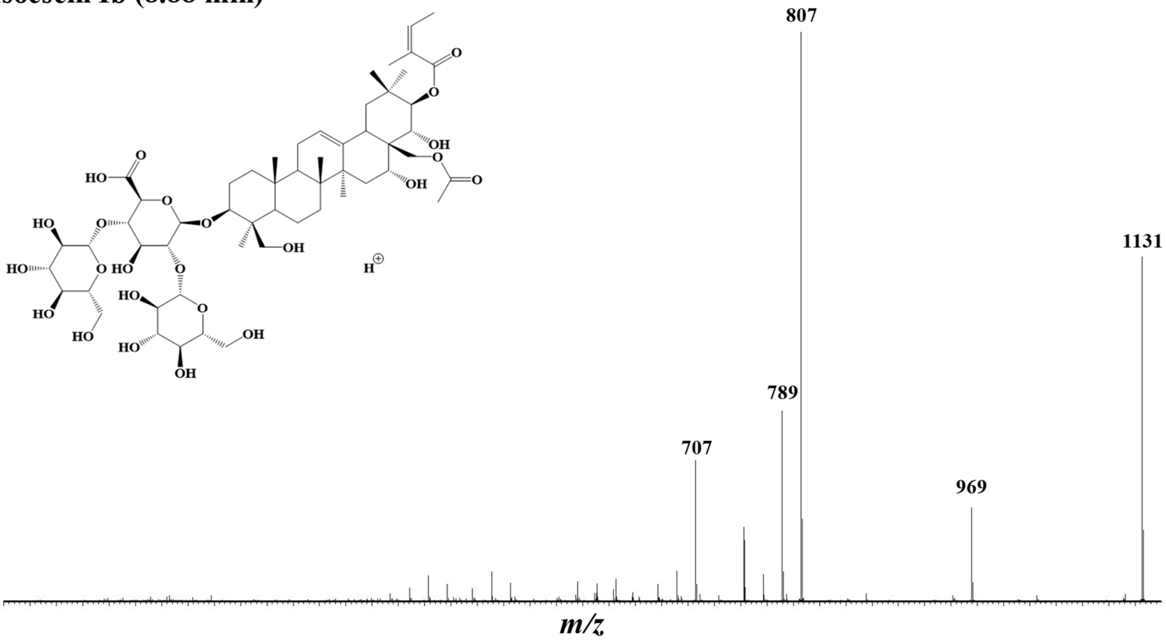

Figure S4: LC-MSMS(+) analysis of Escins I, II and III enriched-extract (EE): CID spectrum (15 eV) recorded for the *m/z* 1131 precursor ions $[M+H]^+$ at 8.88 min retention time (Isoescin Ib)

Transescin Ia (6.59 min)

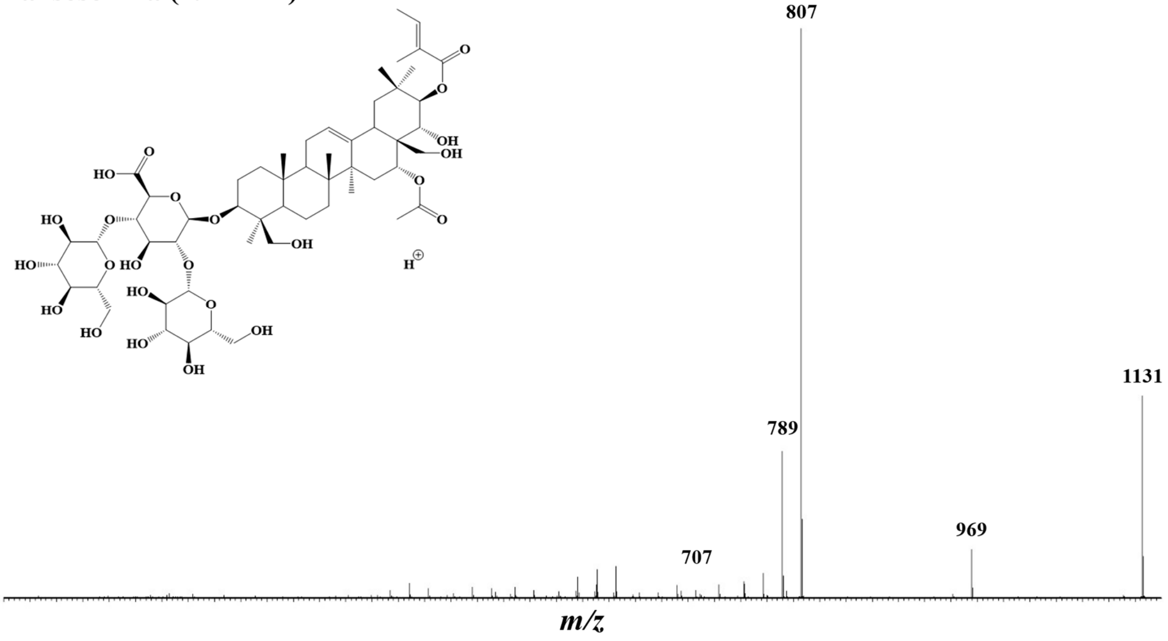

Figure S5: LC-MSMS(+) analysis of Transesterification extract (TE): CID spectrum (15 eV) recorded for the *m/z* 1131 precursor ions $[M+H]^+$ at 6.59 min retention time (Transescin Ia)

Transescin Ib (6.86 min)

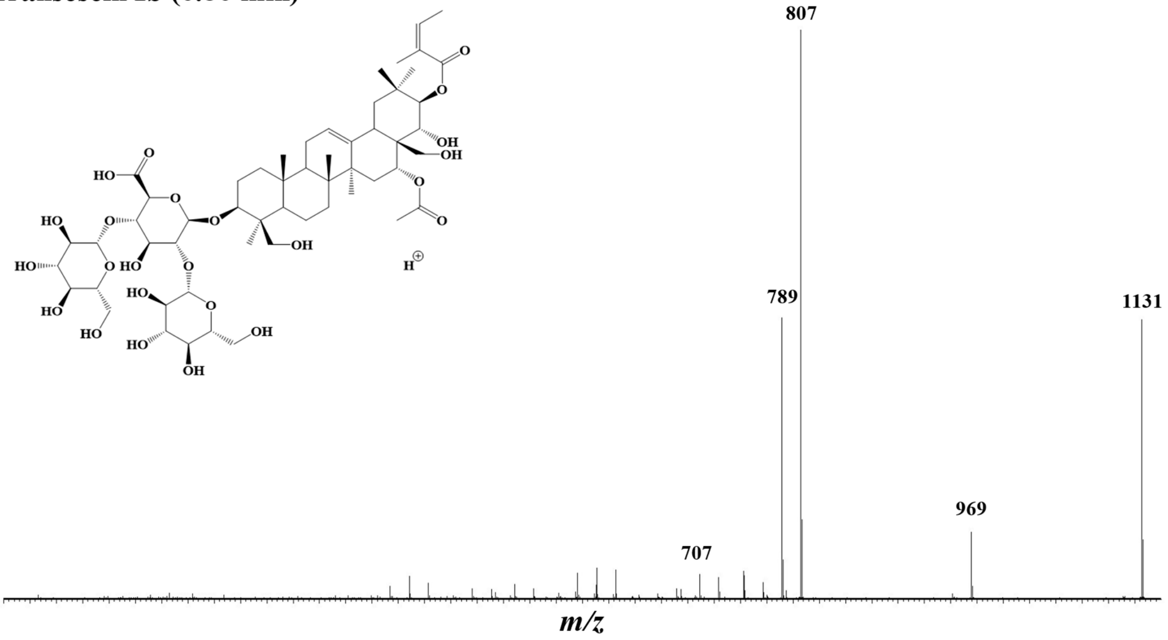

Figure S6: LC-MSMS(+) analysis of Transesterification extract (TE): CID spectrum (15 eV) recorded for the *m/z* 1131 precursor ions $[M+H]^+$ at 6.86 min retention time (Transescin Ib)

Desacylescin I (5.38 min)

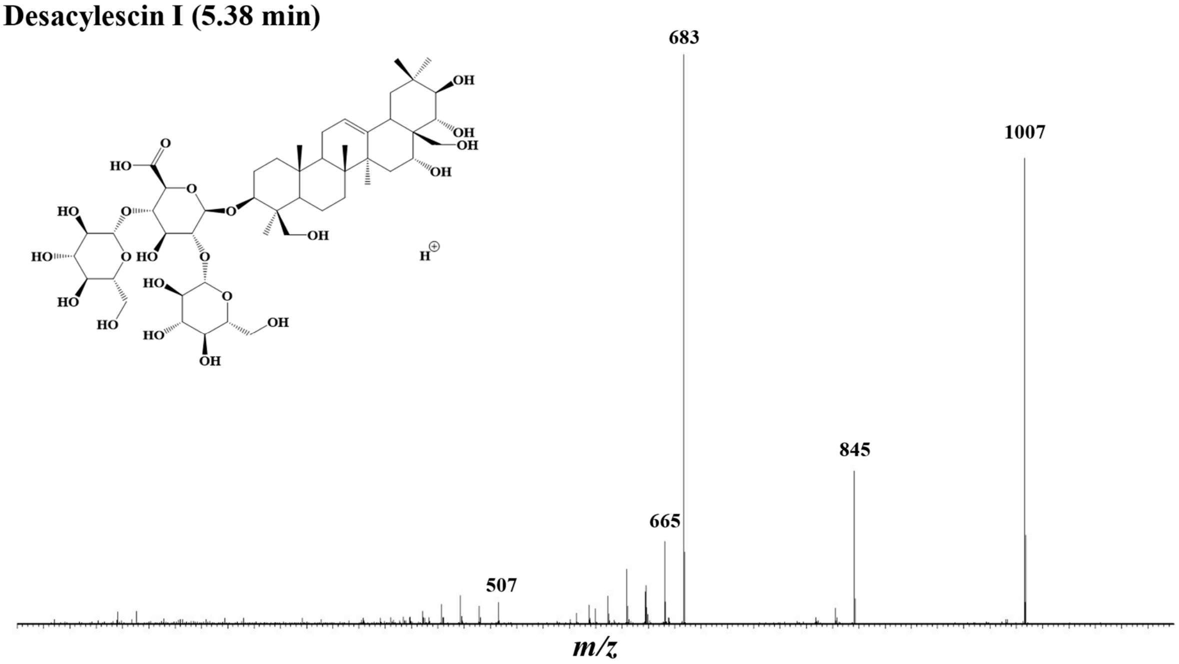

Figure S7: LC-MSMS(+) analysis of Hydrolysis extract (HE): CID spectrum (15 eV) recorded for the *m/z* 1007 precursor ions $[M+H]^+$ at 5.38 min retention time (Desacylescin I)

Escin IIa (7.98 min)

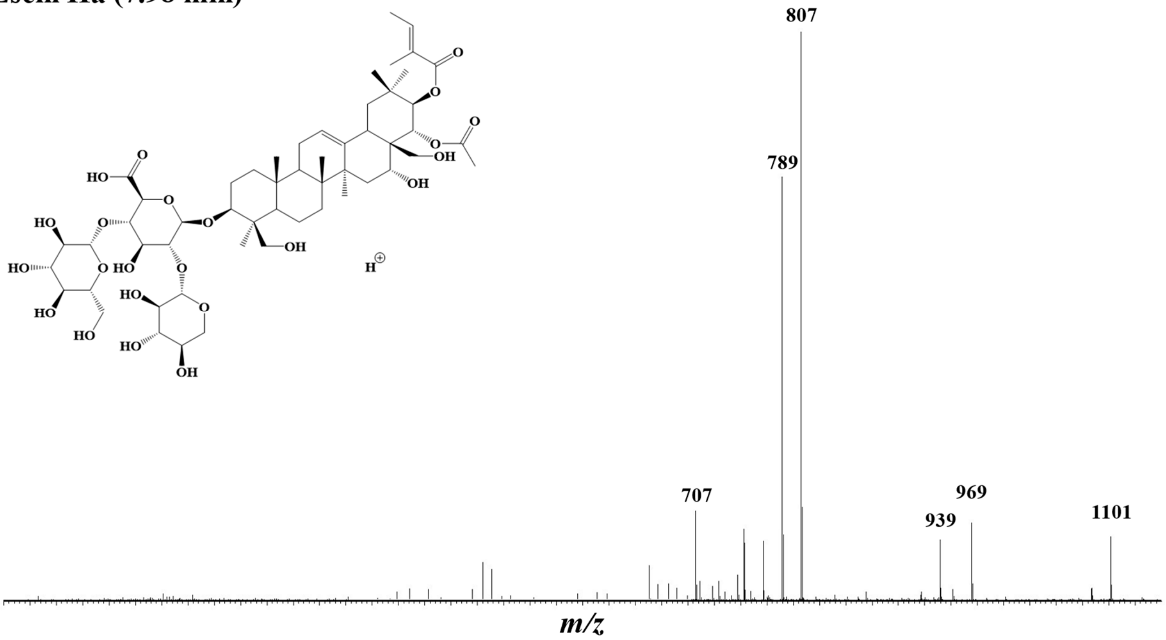

Figure S8: LC-MSMS(+) analysis of Escins I, II and III enriched-extract (EE): CID spectrum (15 eV) recorded for the *m/z* 1101 precursor ions $[M+H]^+$ at 7.98 min retention time (Escin IIa)

Escin IIb (8.28 min)

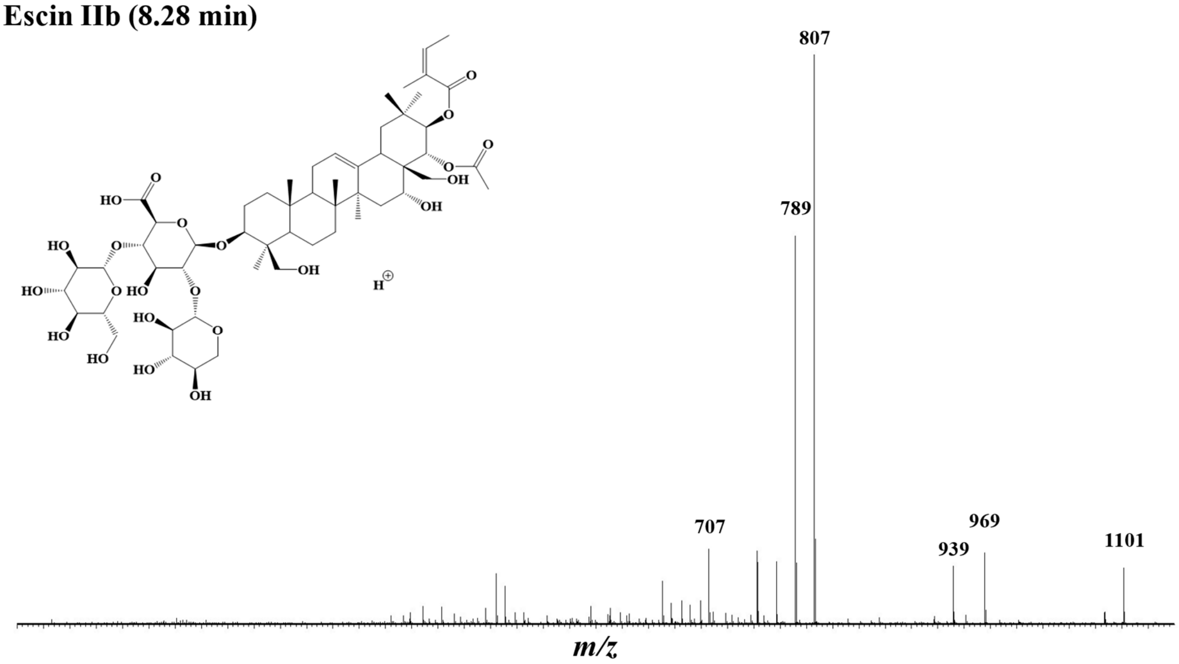

Figure S9: LC-MSMS(+) analysis of Escins I, II and III enriched-extract (EE): CID spectrum (15 eV) recorded for the *m/z* 1101 precursor ions $[M+H]^+$ at 8.28 min retention time (Escin IIb)

Isoescin IIa (8.66 min)

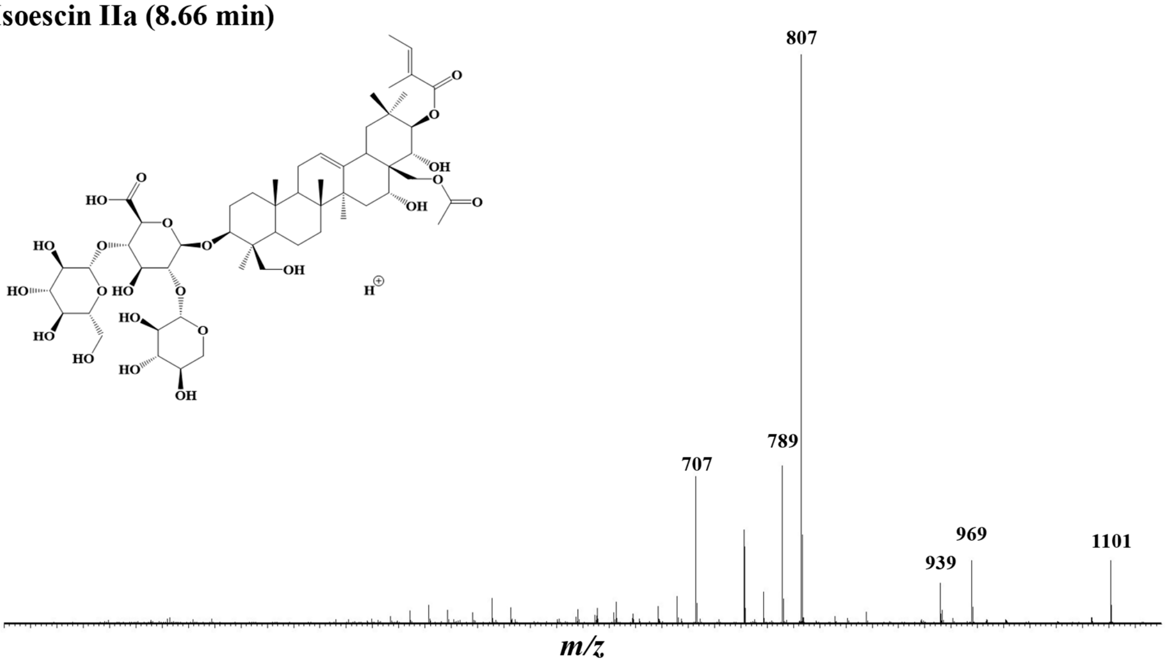

Figure S10: LC-MSMS(+) analysis of Escins I, II and III enriched-extract (EE): CID spectrum (15 eV) recorded for the *m/z* 1101 precursor ions $[M+H]^+$ at 8.66 min retention time (Isoescin IIa)

Isoescin IIb (8.94 min)

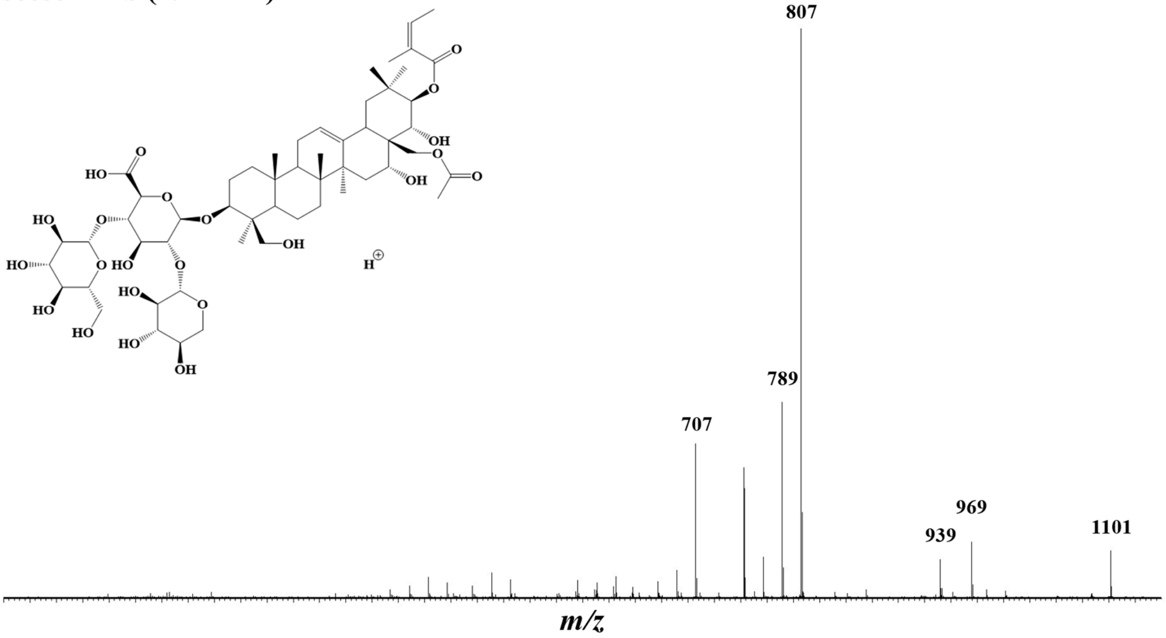

Figure S11: LC-MSMS(+) analysis of Escins I, II and III enriched-extract (EE): CID spectrum (15 eV) recorded for the *m/z* 1101 precursor ions $[M+H]^+$ at 8.94 min retention time (Isoescin IIb)

Transescin IIa (6.58 min)

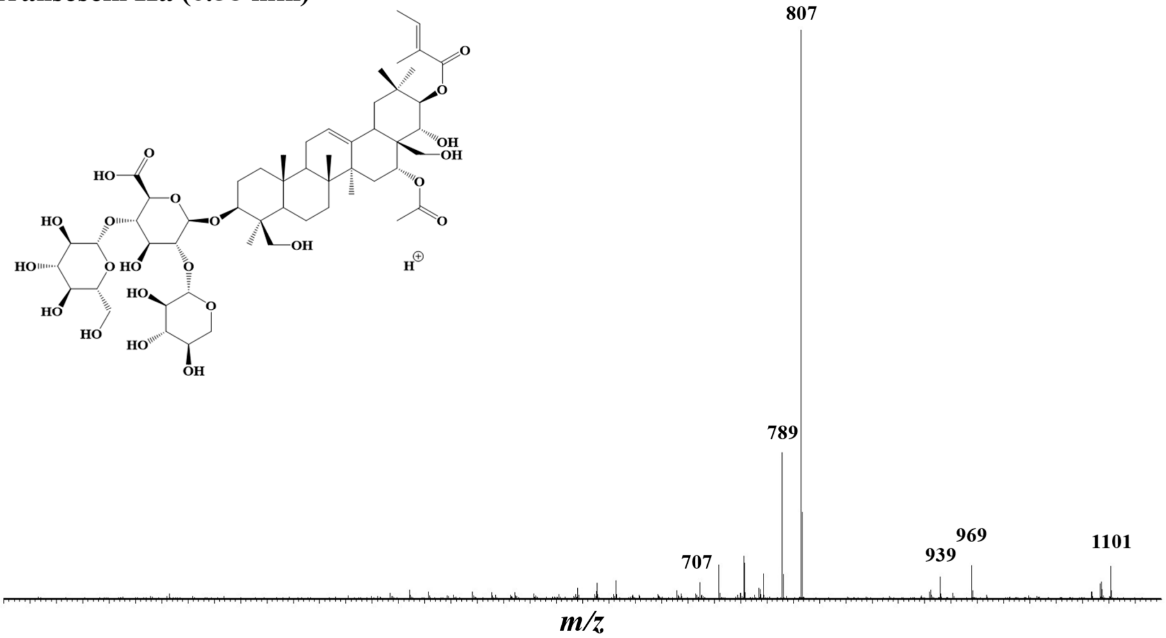

Figure S12: LC-MSMS(+) analysis of Transesterification extract (TE): CID spectrum (15 eV) recorded for the *m/z* 1101 precursor ions $[M+H]^+$ at 6.58 min retention time (Transescin IIa)

Transescin IIb (6.85 min)

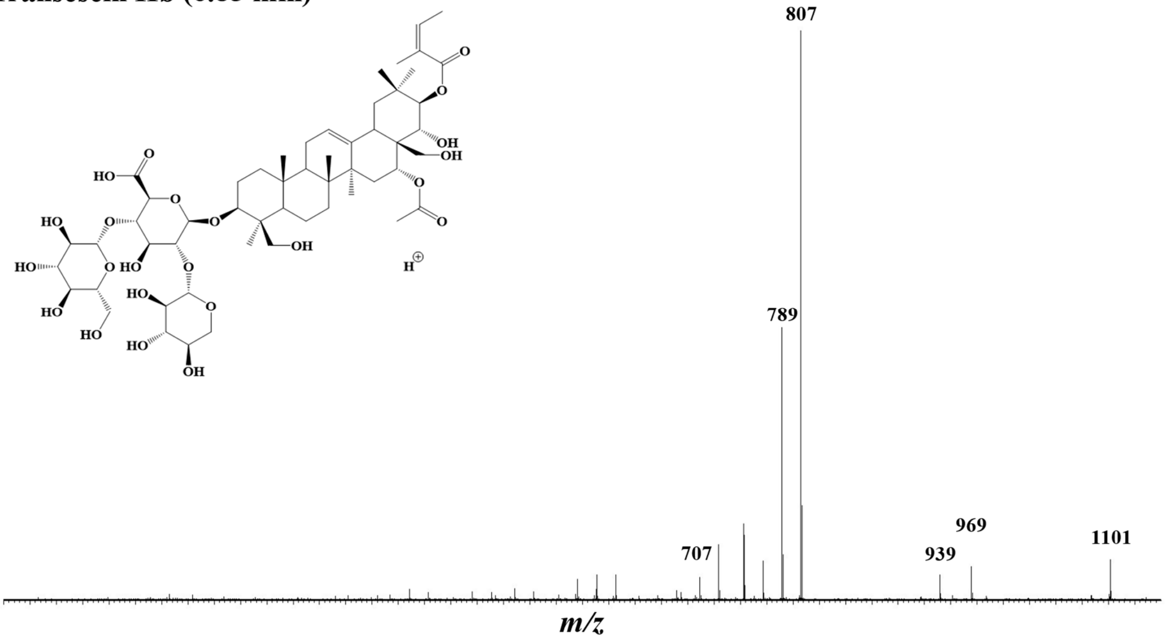

Figure S13: LC-MSMS(+) analysis of Transesterification extract (TE): CID spectrum (15 eV) recorded for the *m/z* 1101 precursor ions $[M+H]^+$ at 6.85 min retention time (Transescin IIb)

Desacylescin II (5.36 min)

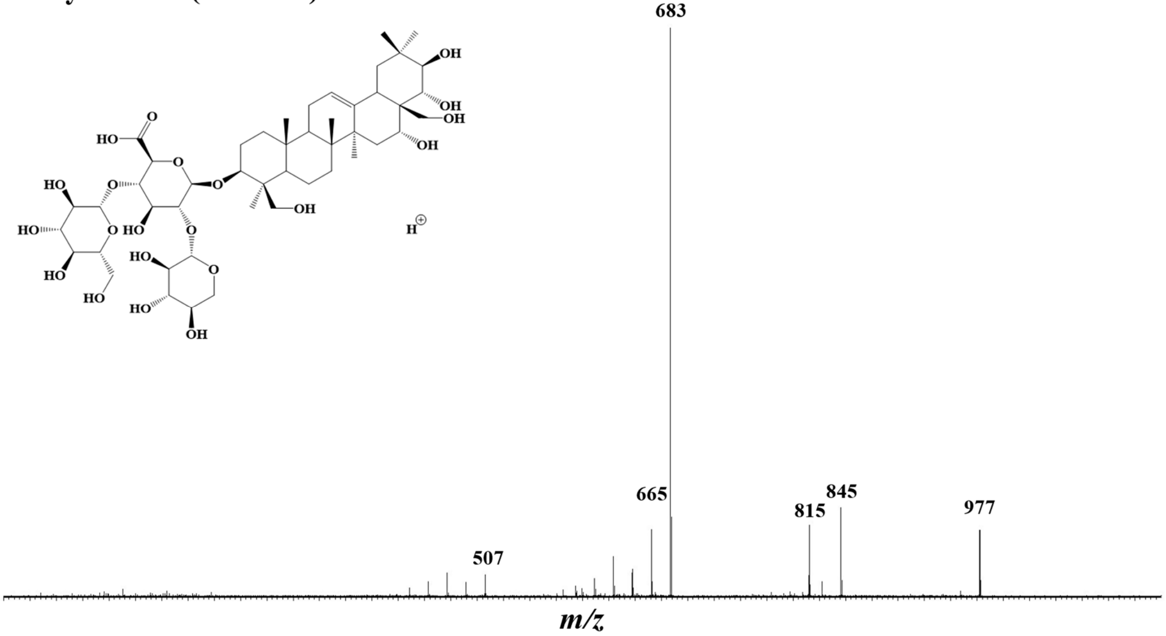

Figure S14: LC-MSMS(+) analysis of Hydrolysis extract (HE): CID spectrum (15 eV) recorded for the *m/z* 977 precursor ions $[M+H]^+$ at 5.36 min retention time (Desacylescin II)

Escin IIIa (8.45 min)

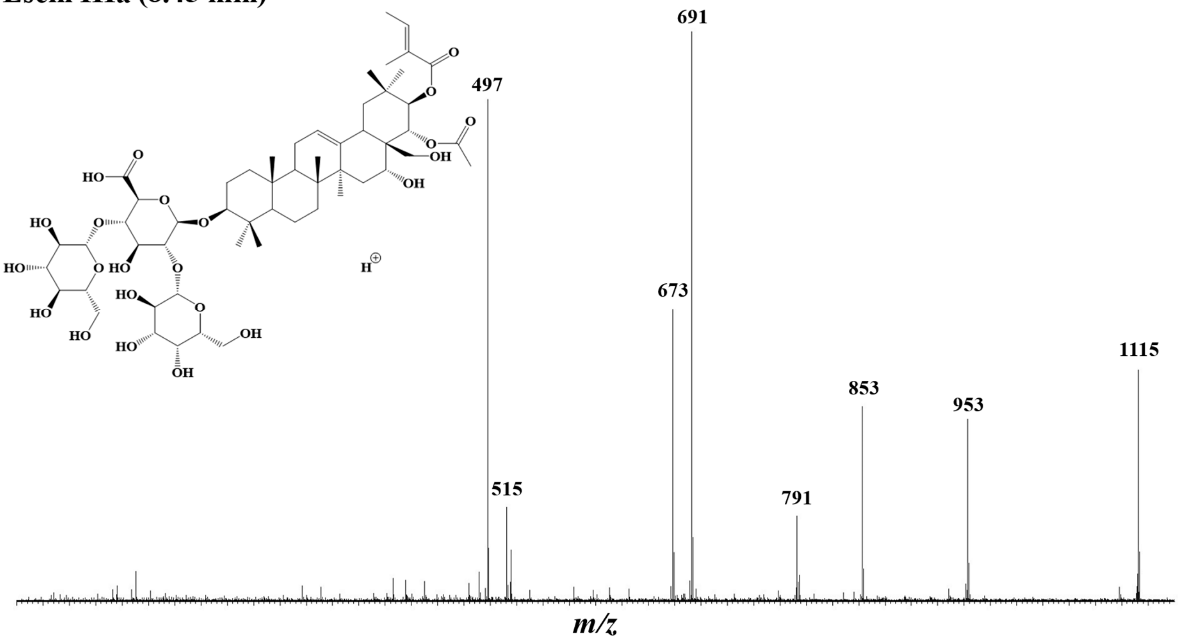

Figure S15: LC-MSMS(+) analysis of Escins I, II and III enriched-extract (EE): CID spectrum (15 eV) recorded for the *m/z* 1115 precursor ions $[M+H]^+$ at 8.45 min retention time (Escin IIIa)

Escin IIIb (8.84 min)

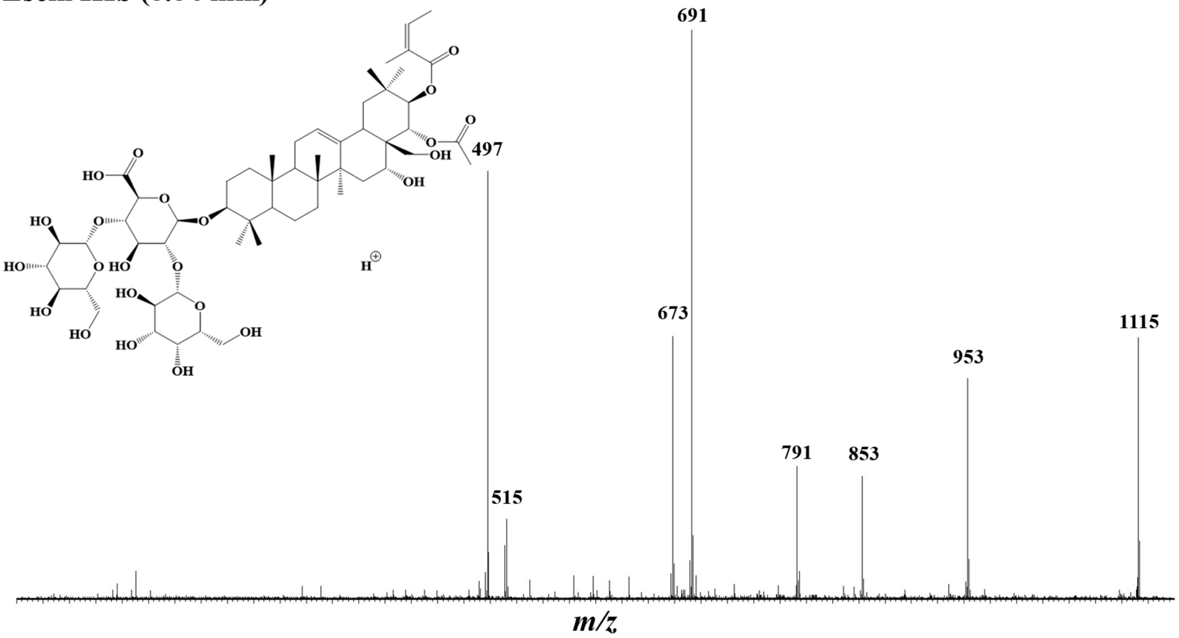

Figure S16: LC-MSMS(+) analysis of Escins I, II and III enriched-extract (EE): CID spectrum (15 eV) recorded for the *m/z* 1115 precursor ions $[M+H]^+$ at 8.84 min retention time (Escin IIIb)

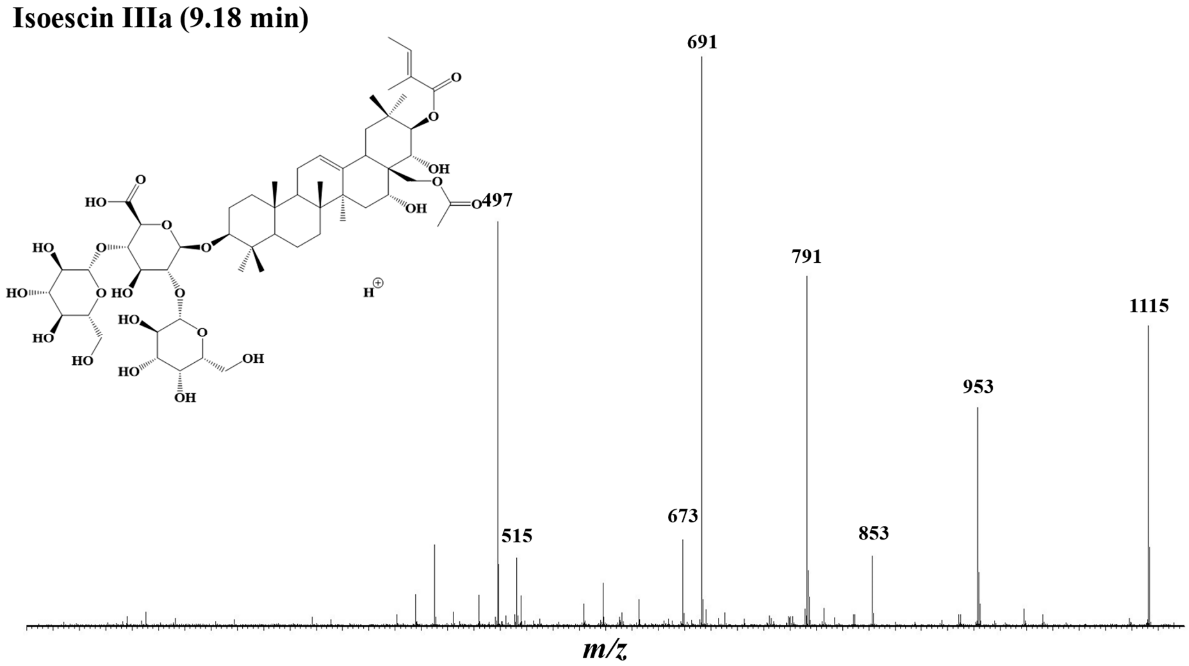

Figure S17: LC-MSMS(+) analysis of Escins I, II and III enriched-extract (EE): CID spectrum (15 eV) recorded for the *m/z* 1115 precursor ions $[M+H]^+$ at 9.18 min retention time (Isoescin IIIa)

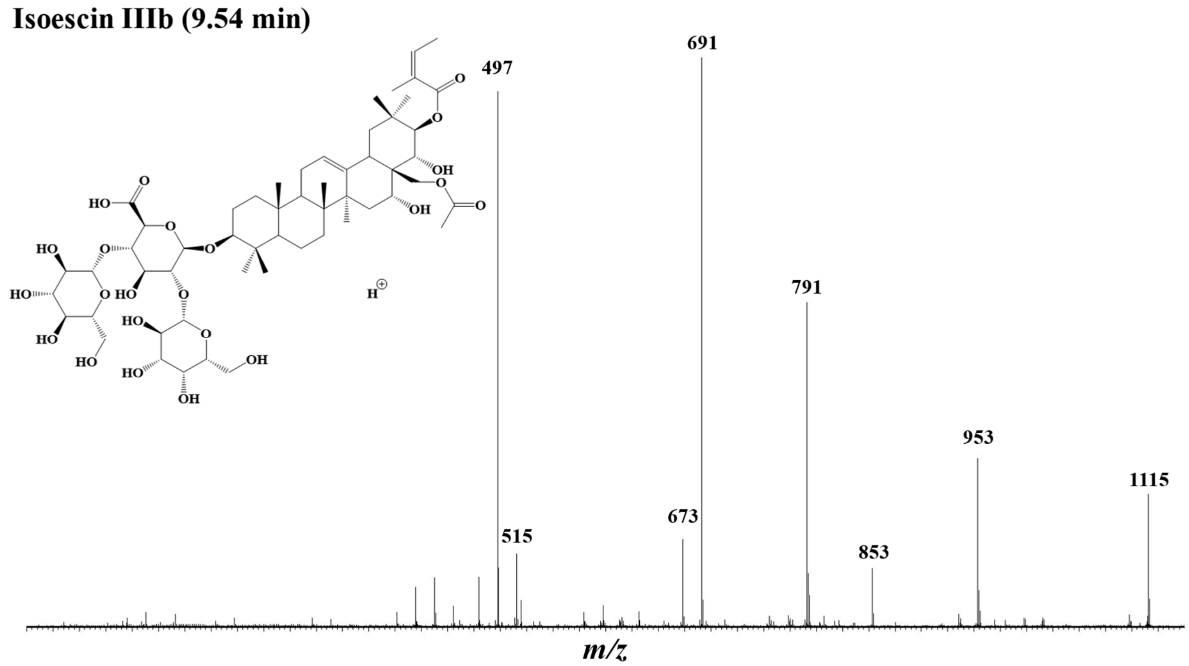

Figure S18: LC-MSMS(+) analysis of Escins I, II and III enriched-extract (EE): CID spectrum (15 eV) recorded for the *m/z* 1115 precursor ions $[M+H]^+$ at 9.54 min retention time (Isoescin IIIb)

Transecsin IIIa (7.00 min)

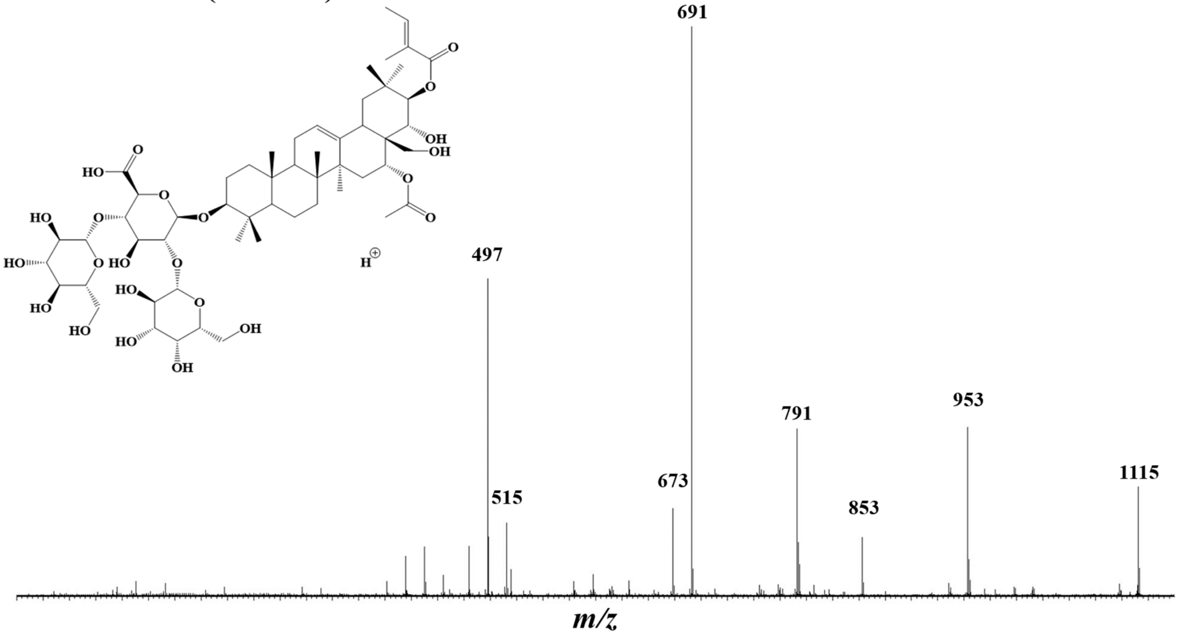

Figure S19: LC-MSMS(+) analysis of Transesterification extract (TE): CID spectrum (15 eV) recorded for the *m/z* 1115 precursor ions $[M+H]^+$ at 7.00 min retention time (Transescin IIIa)

Transescin IIIb (7.27 min)

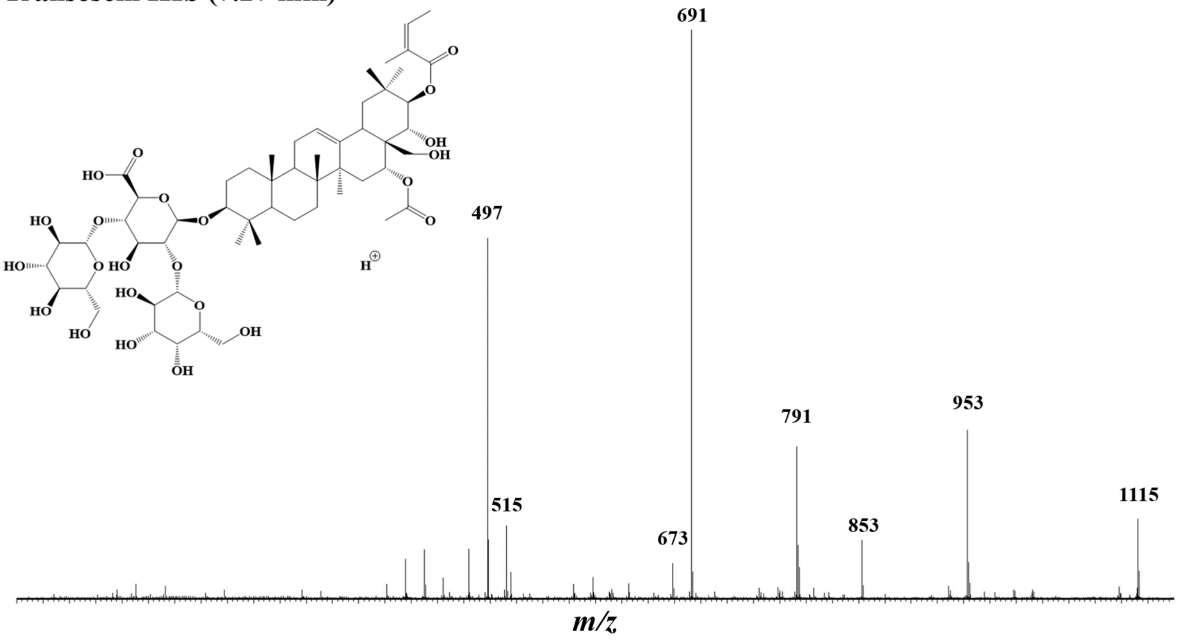

Figure S20: LC-MSMS(+) analysis of Transesterification extract (TE): CID spectrum (15 eV) recorded for the *m/z* 1115 precursor ions $[M+H]^+$ at 7.27 min retention time (Transescin IIIb)

Desacylescin III (5.39 min)

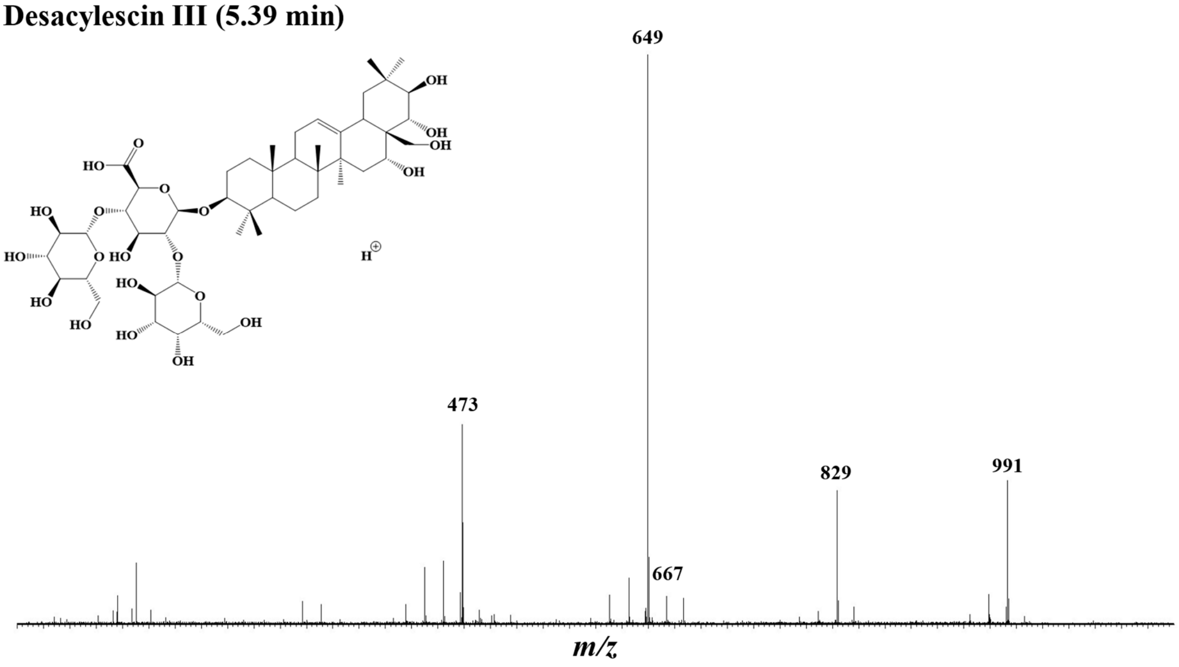

Figure S21: LC-MSMS(+) analysis of Hydrolysis extract (HE): CID spectrum (15 eV) recorded for the *m*/*z* 991 precursor ions $[M+H]^+$ at 5.39 min retention time (Desacylescin III)

Escin IV (6.10 min)

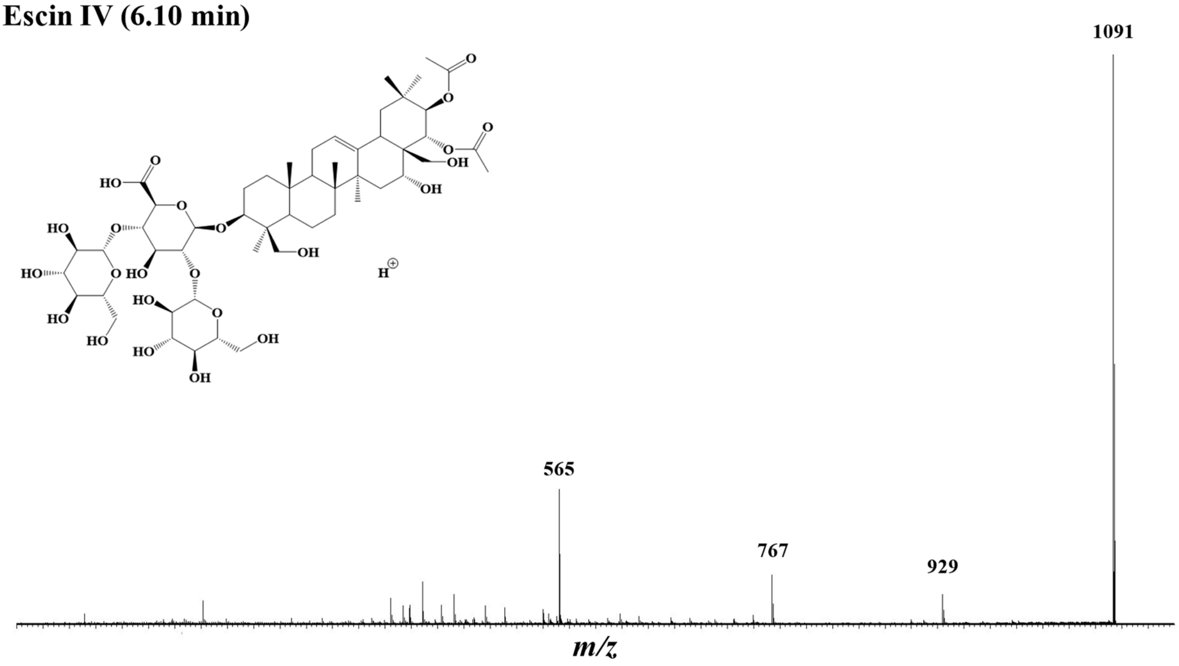

Figure S22: LC-MSMS(+) analysis of Natural extract (NE): CID spectrum (15 eV) recorded for the *m*/*z* 1091 precursor ions $[M+H]^+$ at 6.10 min retention time (Escin IV)

Isoescin IV (6.56 min)

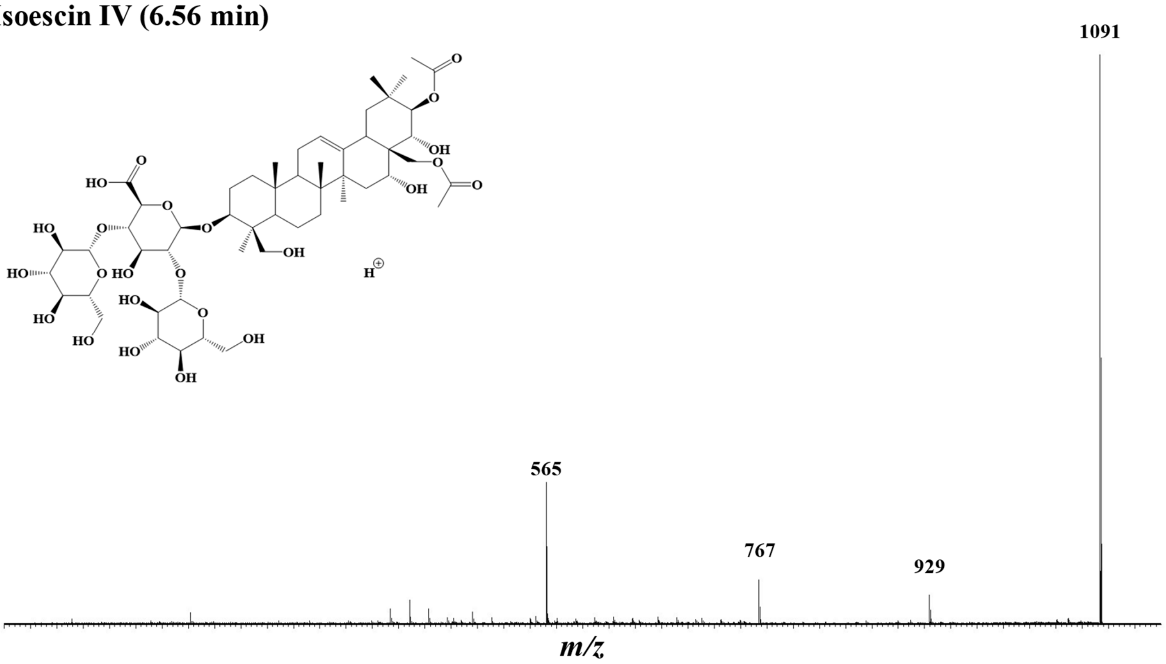

Figure S23: LC-MSMS(+) analysis of Natural extract (NE): CID spectrum (15 eV) recorded for the *m/z* 1091 precursor ions $[M+H]^+$ at 6.56 min retention time (Isoescin IV)

Escin V (7.71 min)

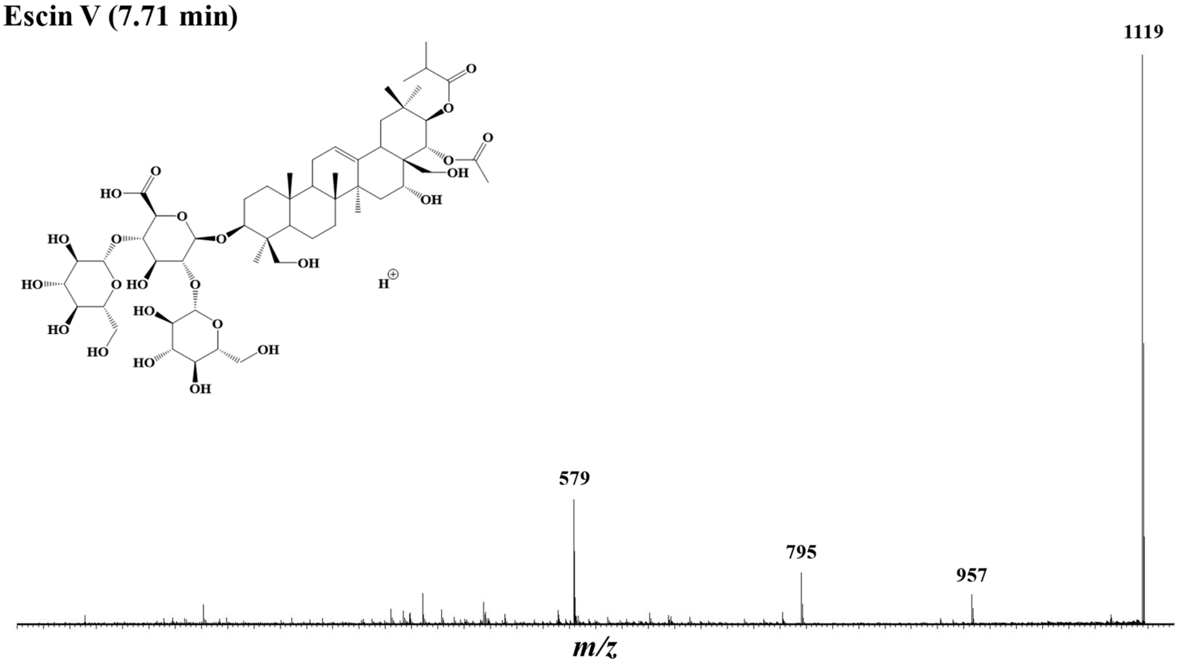

Figure S24: LC-MSMS(+) analysis of Natural extract (NE): CID spectrum (15 eV) recorded for the *m/z* 1119 precursor ions $[M+H]^+$ at 7.71 min retention time (Escin V)

Isoescin V (8.20 min)

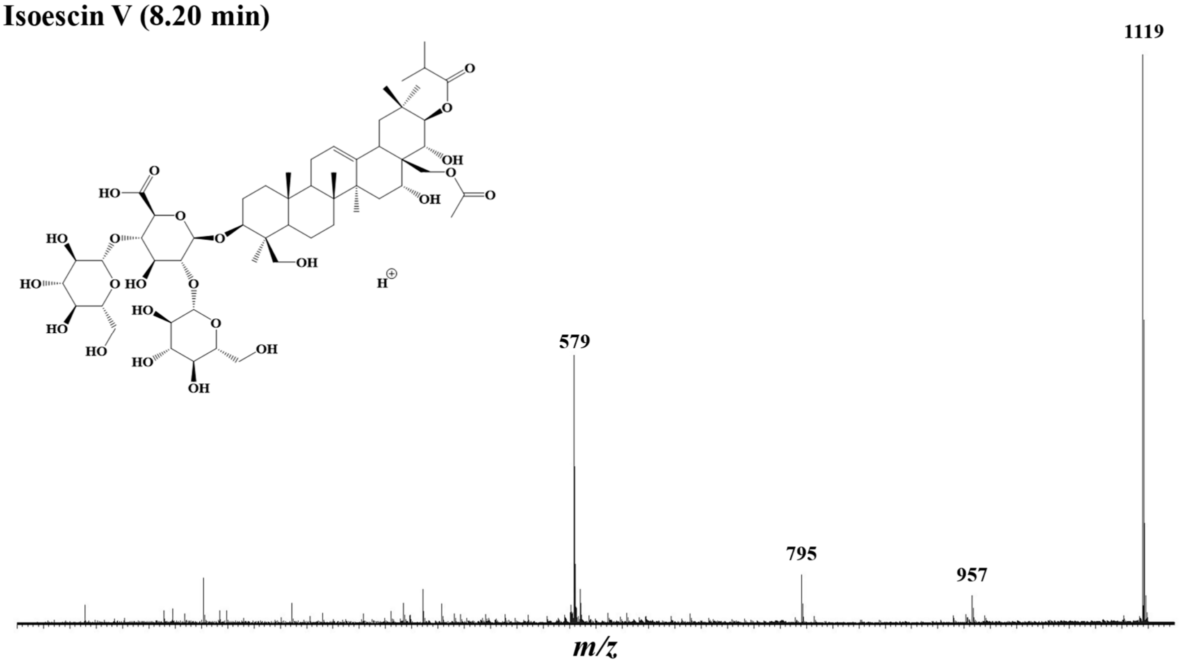

Figure S25: LC-MSMS(+) analysis of Natural extract (NE): CID spectrum (15 eV) recorded for the *m/z* 1119 precursor ions $[M+H]^+$ at 8.20 min retention time (Isoescin V)

Escin VI (8.62 min)

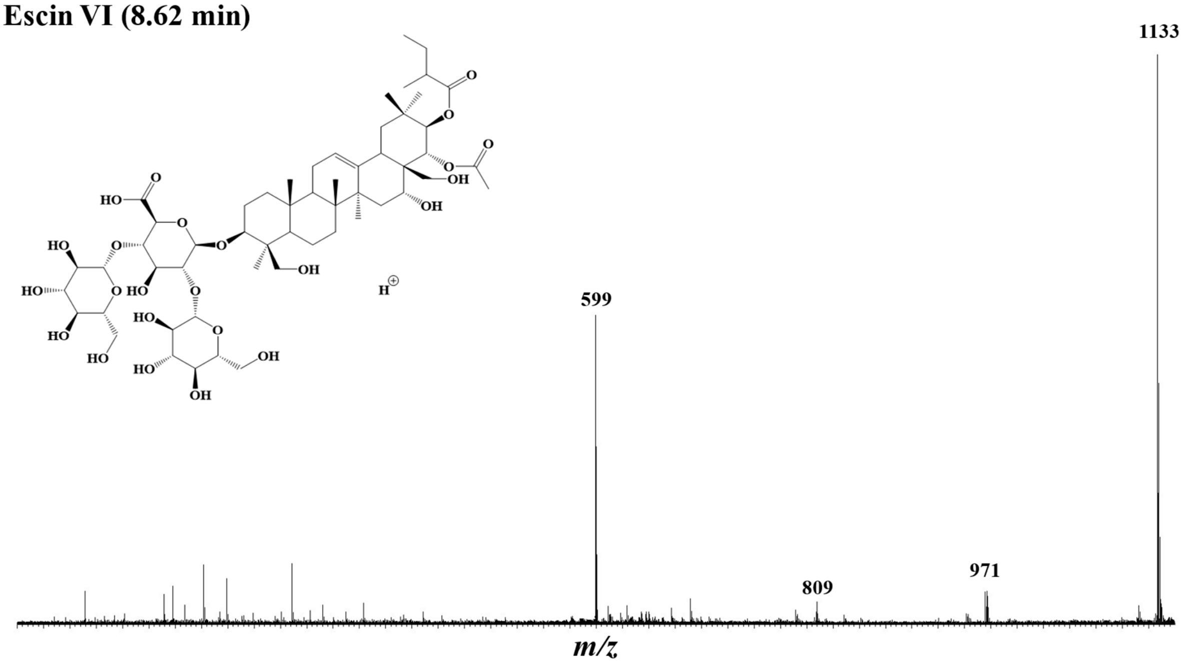

Figure S26: LC-MSMS(+) analysis of Natural extract (NE): CID spectrum (15 eV) recorded for the *m/z* 1133 precursor ions $[M+H]^+$ at 8.62 min retention time (Escin VI)

Isoescin VI (9.47 min)

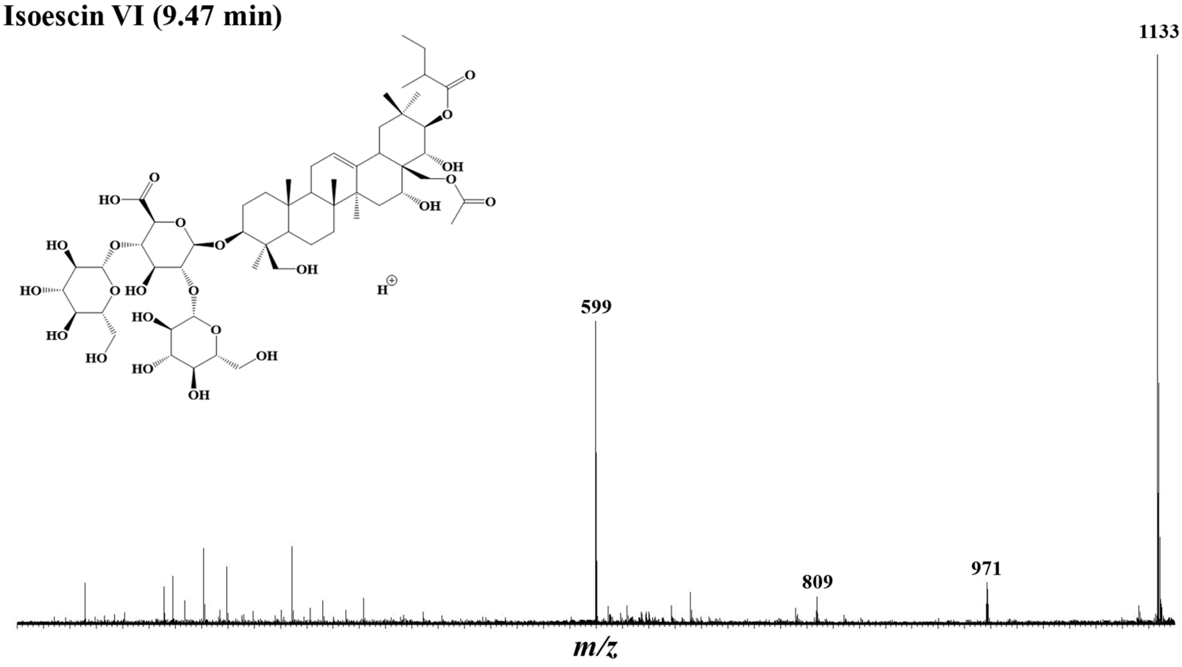

Figure S27: LC-MSMS(+) analysis of Natural extract (NE): CID spectrum (15 eV) recorded for the *m/z* 1133 precursor ions $[M+H]^+$ at 9.47 min retention time (Isoescin VI)

Escin VII (6.12 min)

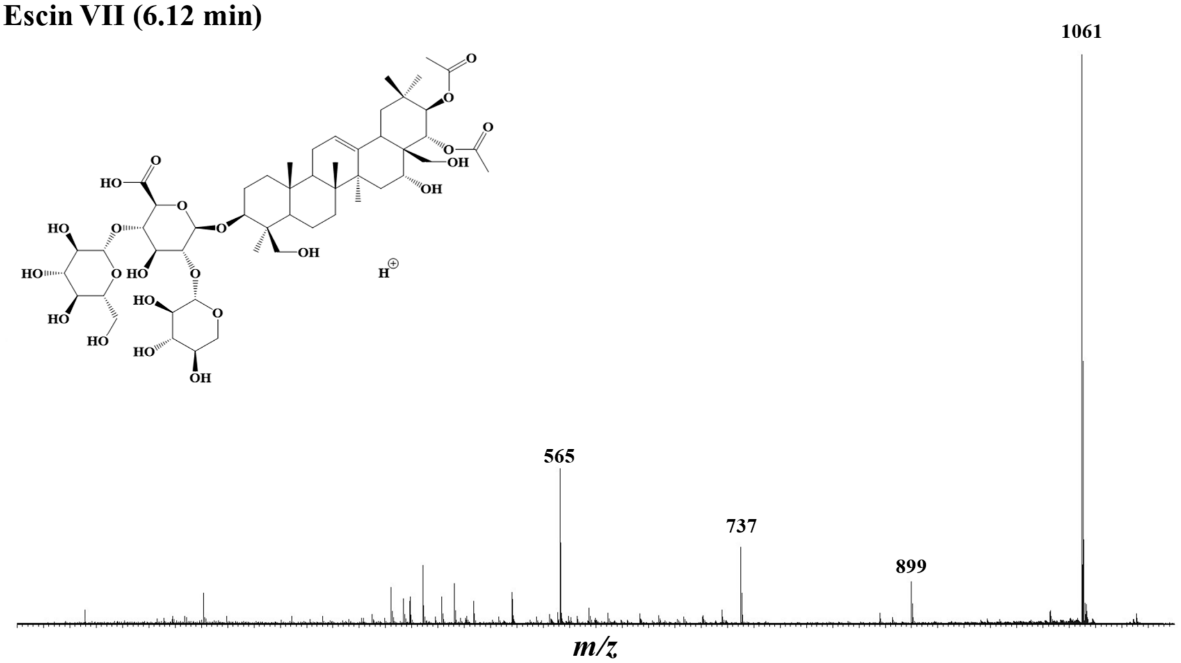

Figure S28: LC-MSMS(+) analysis of Natural extract (NE): CID spectrum (15 eV) recorded for the *m/z* 1061 precursor ions $[M+H]^+$ at 6.12 min retention time (Escin VII)

Isoescin VII (6.59 min)

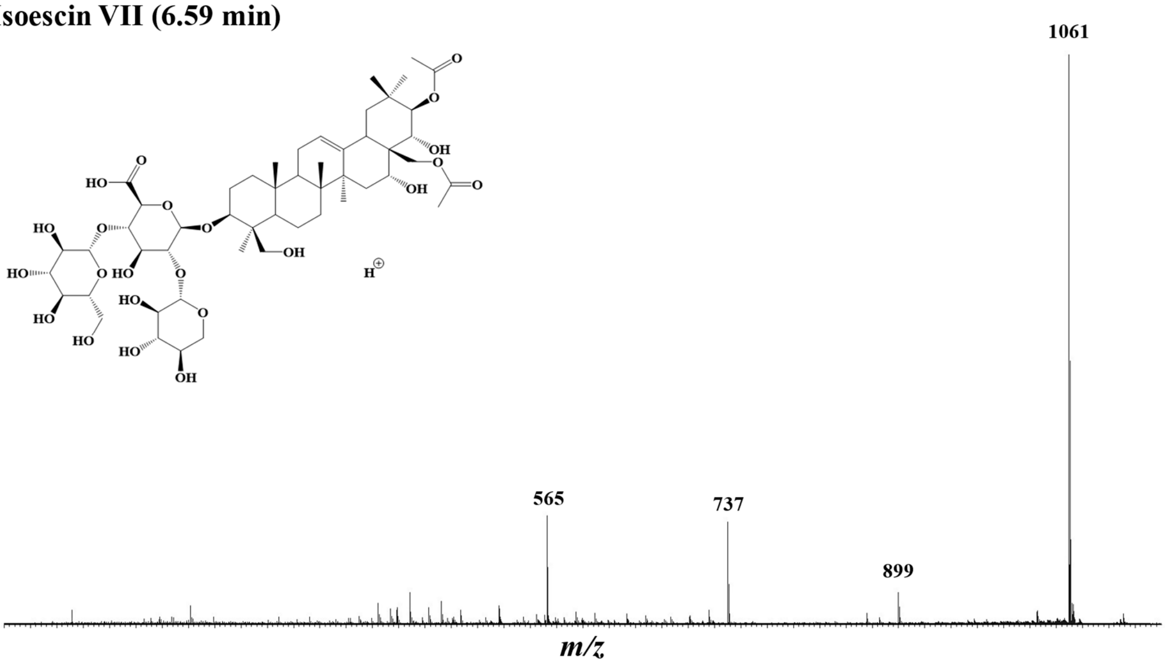

Figure S29: LC-MSMS(+) analysis of Natural extract (NE): CID spectrum (15 eV) recorded for the *m/z* 1061 precursor ions $[M+H]^+$ at 6.59 min retention time (Isoescin VII)

Escin VIII (7.42 min)

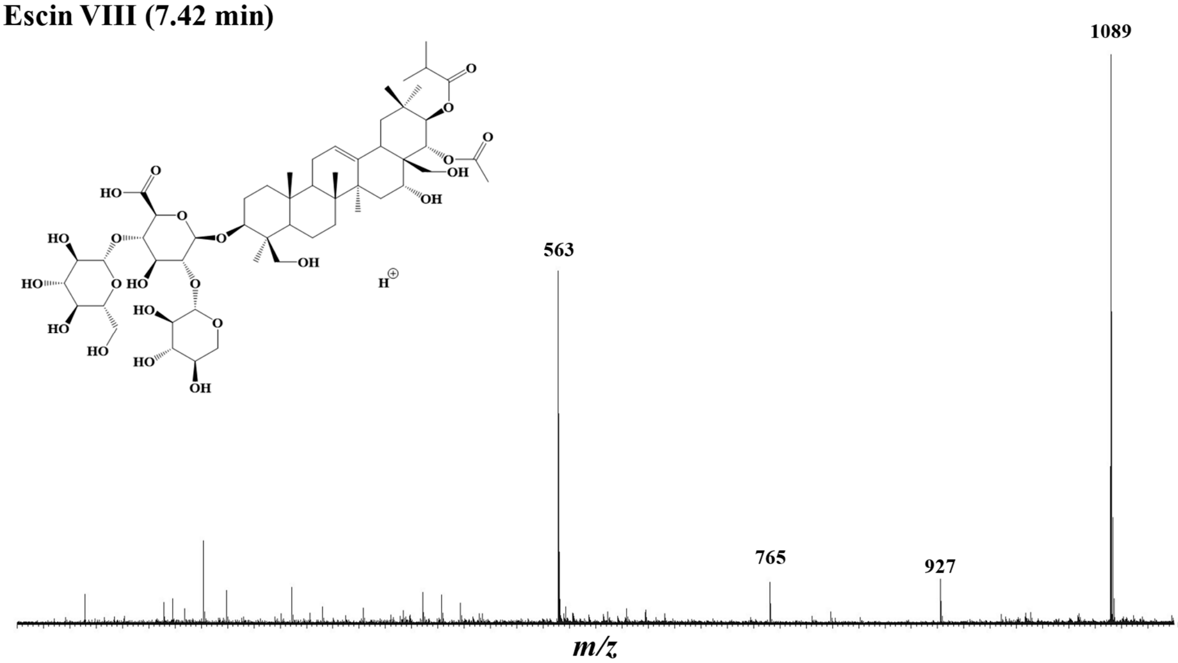

Figure S30: LC-MSMS(+) analysis of Natural extract (NE): CID spectrum (15 eV) recorded for the *m/z* 1089 precursor ions $[M+H]^+$ at 7.42 min retention time (Escin VIII)

Isoescin VIII (7.64 min)

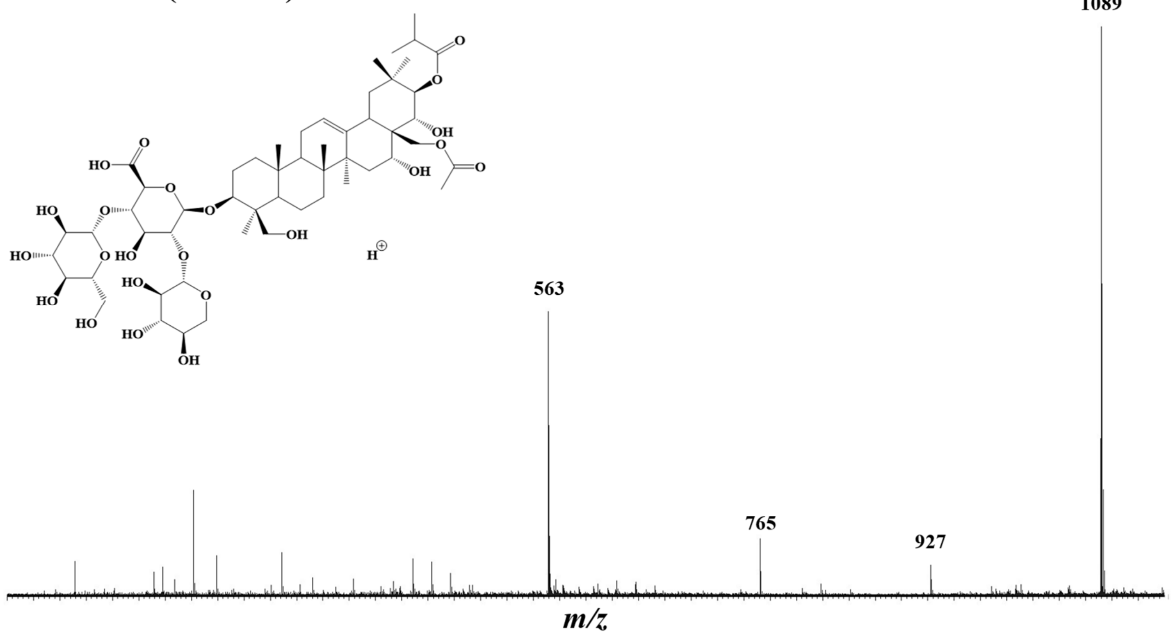

Figure S31: LC-MSMS(+) analysis of Natural extract (NE): CID spectrum (15 eV) recorded for the *m/z* 1089 precursor ions $[M+H]^+$ at 7.64 min retention time (Isoescin VIII)

Escin IX (6.44 min)

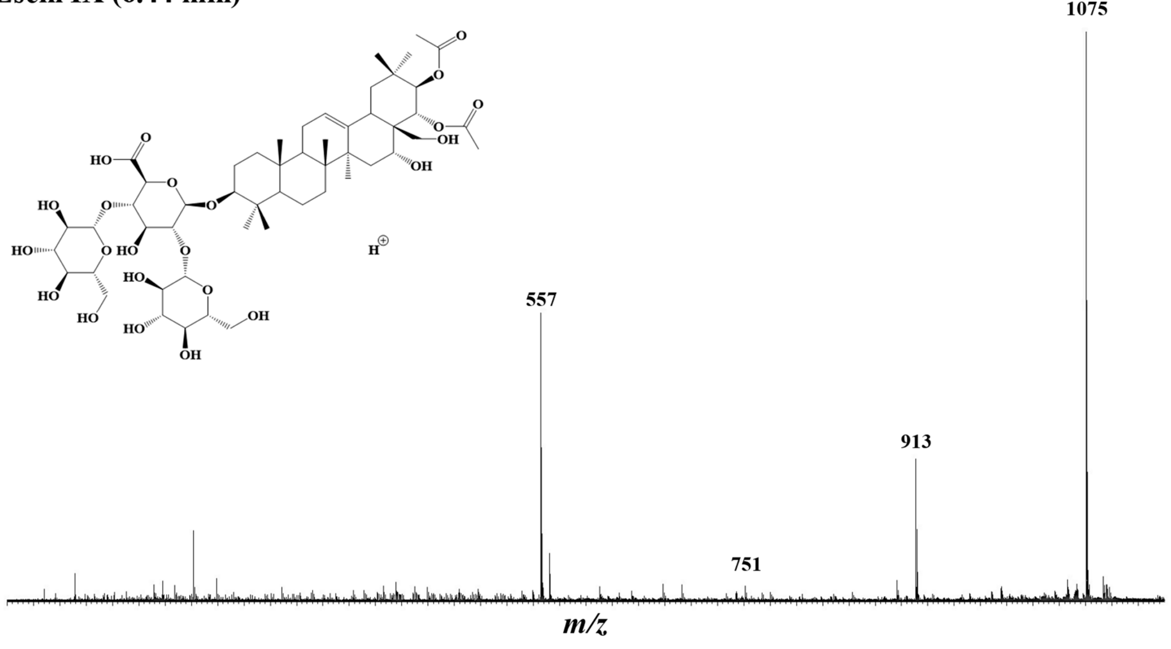

Figure S32: LC-MSMS(+) analysis of Natural extract (NE): CID spectrum (15 eV) recorded for the *m/z* 1075 precursor ions $[M+H]^+$ at 6.44 min retention time (Escin IX)

Isoescin IX (6.83 min)

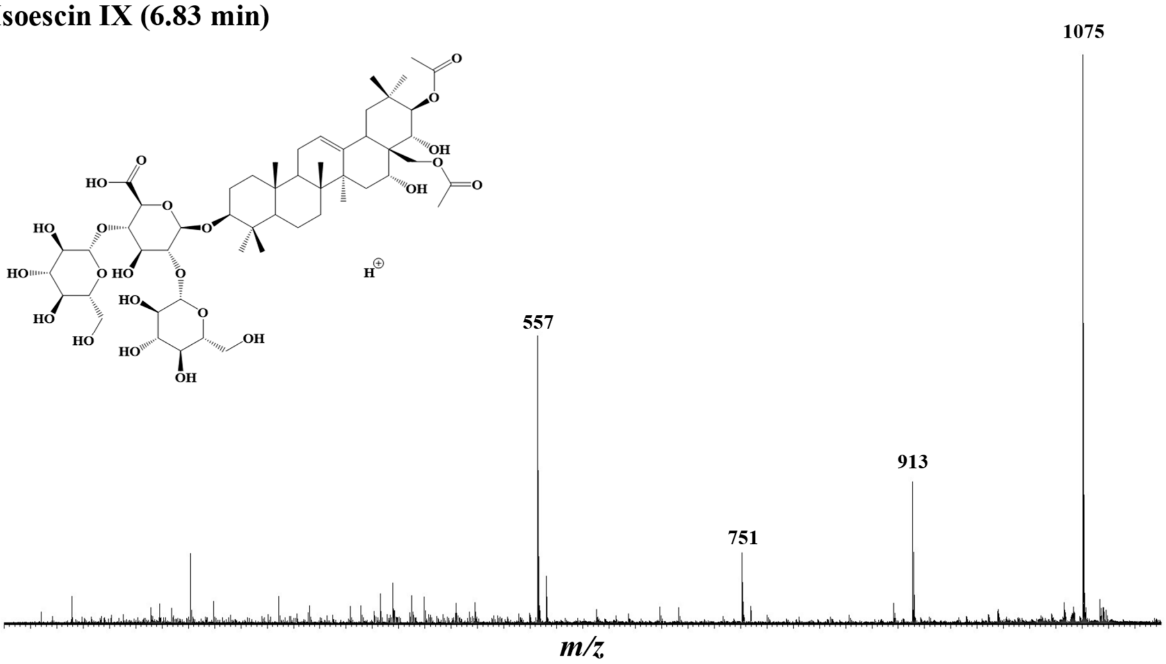

Figure S33: LC-MSMS(+) analysis of Natural extract (NE): CID spectrum (15 eV) recorded for the *m/z* 1075 precursor ions $[M+H]^+$ at 6.83 min retention time (Isoescin IX)
